# Molecular, parasitological and clinical diagnosis of female genital schistosomiasis: A diagnostic yield and comparative accuracy study using latent class analysis

**DOI:** 10.1007/s00436-026-08753-8

**Published:** 2026-09-18

**Authors:** Adela Ngwewondo, Clarisse Engowei Mbah, Kingsley Ombaku Sama, Godlove Bunda Wepnje, Forgu Esemu Livo, Elvis Monya, Roger Somo Moyou

**Affiliations:** 1 Institute of Medical Research and Medicinal Plants Studies, P.O Box: 13033, Yaounde, Cameroon; 2 Faculty of Health Sciences, University of Bamenda, P.O. Box 39, Bambili, Cameroon; 3 Saint Monica University Higher institute, P.O Box 132, Buea, Cameroon; 4 Faculty of Science, University of Buea, P.O Box 63, Buea, Cameroon; 5 Faculty of Medicine and Biomedical Sciences, University of Yaounde I, P.O Box 1364, Yaounde, Cameroon

**Keywords:** Real-time PCR, Female genital schistosomiasis, Urine egg microscopy, Colposcopy, Diagnostic accuracy, Latent class analysis

## Abstract

Background: Female genital schistosomiasis (FGS) is a neglected manifestation of Schistosoma haematobium infection whose diagnosis is restricted by the absence of a flawless "gold standard" diagnostic tool. This study compared the yield, accuracy, performance of polymerase chain reaction (PCR), urine egg microscopy, and colposcopy. Methods: A cross-sectional study was conducted among 143 female participants from S. haematobium-endemic communities in the West Region of Cameroon between July and August 2024. Participants underwent urine microscopy, genital swab PCR, and colposcopy, and a two-class latent class analysis model was developed using the R package poLCA. Results: PCR yielded the highest positivity rate (73.4%), followed by urine egg microscopy (29.4%) and colposcopy/Visual FGS (23.08%). Among women with visual FGS, 68.75% were PCR-positive and had a lower median Ct value (29.6) than PCR-negative women (32.8) by SYBR Green PCR. Within the LCA framework, PCR provided a baseline sensitivity of 100.00% (95% CI: 94.85%–100.00%) and a specificity of 95.20% (95% CI: 91.88%–98.52%). urine egg microscopy and colposcopy/visual FGS served as highly specific rule-out tools with specificities of 83.32% (95% CI: 62.75%–100.00%) and 83.33% (95% CI: 62.76%–100.00%), despite their lower sensitivities of 29.06% (95% CI: 17.5%–40.62%) and 38.27% (95% CI: 29.92%–46.62%) respectively. Conclusion: The LCA model confirmed that a negative result from traditional tools cannot rule out infection necessitating the use of complementary diagnostic tools. However, clinicians should interpret positive PCR outcomes with care, as molecular detection cannot distinguish between active infection, recent parasite exposure, or residual DNA from past infections.

**Supplementary Information:**

The online version contains supplementary material available at https://doi.org/10.1007/s00436-026-08753-8.

**Figure:**
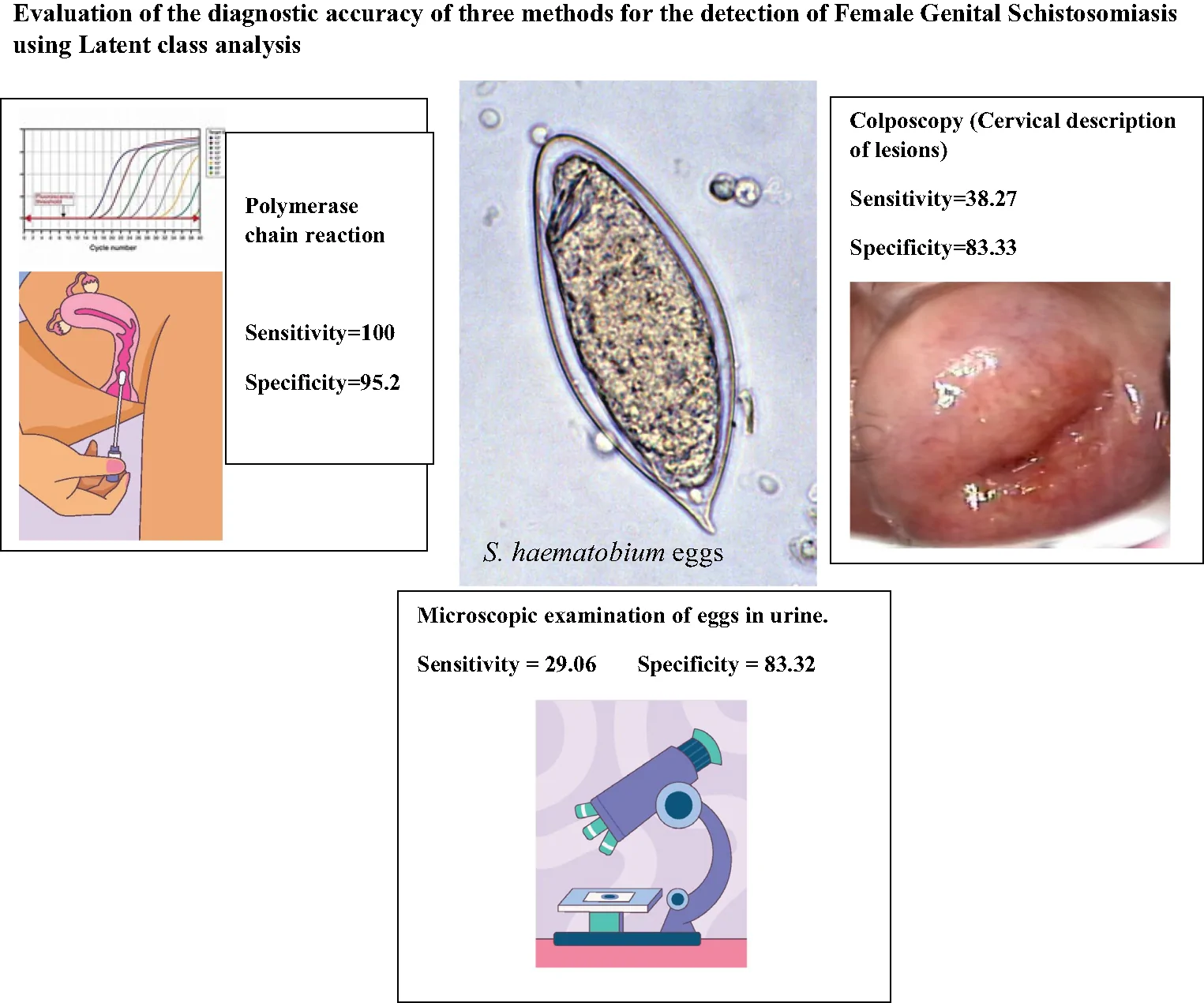

## Introduction

Female genital schistosomiasis (FGS) is a chronic manifestation of infection with *S. haematobium* and represents one of the most neglected reproductive health conditions affecting women and girls in schistosomiasis-endemic regions (Bustinduy et al. 2022; WHO 2022; Mberu et al. 2025). Although preventive chemotherapy has substantially reduced the burden of schistosomiasis in many countries, transmission remains widespread across sub-Saharan Africa (90% of global cases) due to continuous re-exposure to contaminated water bodies(Colley et al. 2014; WHO 2022). Chronic deposition of *S. haematobium* eggs within the female genital tract induces persistent granulomatous inflammation, fibrosis and abnormal vascularization, resulting in lesions of the cervix, vagina, vulva and upper reproductive tract(Kjetland et al. 2014; Bustinduy et al. 2022). These pathological changes have been associated with genital pain, abnormal vaginal discharge, contact bleeding, dyspareunia, infertility, adverse pregnancy outcomes, and an increased susceptibility to sexually transmitted infections, including HIV and human papillomavirus (HPV) (Kjetland et al. 2012; Wall et al., 2018; Bustinduy et al. 2022; Mberu et al. 2025). Recent estimates suggest that between 20 and 56 million women and girls may be living with FGS, although the true burden is likely underestimated because of limited surveillance and underdiagnosis (Poggensee and Feldmeier, 2001; Mberu et al. 2025).

Cameroon remains endemic for urogenital schistosomiasis despite decades of control efforts. Persistent transmission has been reported in several regions, particularly among populations with frequent contact with freshwater bodies that support intermediate snail hosts (Mewabo et al. 2017; Njunda et al. 2017). National control programmes have primarily focused on preventive chemotherapy among school-aged children, while adult women remain insufficiently covered despite their continued risk of infection and chronic genital disease (Tchuem Tchuenté et al. 2017; Masong et al. 2021; WHO 2022). Although the epidemiology of urinary schistosomiasis has been well documented in Cameroon, the prevalence and burden of FGS remain poorly characterized because routine surveillance and sensitive diagnostic approaches are rarely implemented (Bustinduy et al. 2022; Mberu et al. 2025). This knowledge gap limits accurate estimates of disease burden and hinders the integration of FGS into reproductive health programs.

Urine microscopy remains the reference parasitological method for diagnosing urogenital schistosomiasis by demonstrating the active excretion of *S. haematobium* eggs in urine, thereby providing evidence of ongoing infection and transmission (Utzinger et al. 2015; Weerakoon et al. 2015). The development of FGS depends on the deposition of parasite eggs within genital tissues, and women with established genital disease may exhibit low or intermittent urinary egg excretion or even negative urine microscopy despite significant genital pathology (Kjetland et al. 2012; Bustinduy et al. 2022). Although urine microscopy does not directly diagnose FGS, it contributes important information on the underlying infection process and complements molecular and clinical assessments of genital disease. Accurate diagnosis of FGS remains one of the greatest challenges to disease control because no single diagnostic method can reliably identify all infected women (Utzinger et al. 2015; Weerakoon et al. 2015; Bustinduy et al. 2022). Real-time polymerase chain reaction (PCR) performed on genital specimens enables sensitive detection of *S. haematobium* DNA and has demonstrated improved performance for identifying genital infection, particularly among women with low parasite burdens (Randrianasolo et al. 2015; Utzinger et al. 2015). Colposcopy, in contrast, permits direct visualization of the characteristic lesions of FGS, including sandy patches, rubbery papules and abnormal blood vessels, thereby providing clinical evidence of chronic genital pathology (Kjetland et al. 2014; Norseth et al. 2014). However, lesion recognition depends heavily on examiner expertise, specialized equipment and standardized interpretation, limiting its applicability in many endemic settings (Norseth et al. 2014; Valls et al. 2023). Furthermore, the appearance of FGS lesions may overlap with cervical abnormalities caused by sexually transmitted infections or cervical neoplasia, thereby reducing diagnostic specificity (Norseth et al. 2014; Bustinduy et al. 2022).

The absence of a universally accepted gold standard for FGS has complicated the evaluation of existing and emerging diagnostic methods. Conventional estimates of diagnostic sensitivity and specificity assume that the reference test accurately classifies disease status, an assumption that is inappropriate when all available tests measure different biological aspects of infection.

Latent class analysis (LCA) has emerged as a robust statistical approach for estimating diagnostic accuracy when no perfect reference standard exists. LCA assumes that the observed results from multiple imperfect tests are driven by an unobserved (latent) disease status and uses the pattern of agreement and disagreement among tests to estimate the probability that an individual truly belongs to the infected or non-infected class, while simultaneously estimating the sensitivity and specificity of each diagnostic test (Hui and Walter 1980; Joseph et al. 1995; Dendukuri and Joseph 2001) The method has been widely applied to evaluate diagnostic tests for infectious and neglected tropical diseases, including schistosomiasis, tuberculosis and malaria, where no single test adequately reflects true infection status (Dendukuri and Joseph 2001; Ibironke et al. 2012; Rachid et al. 2024) allowing unbiased estimation of test performance and disease prevalence. In the context of FGS, where urine microscopy, genital PCR and colposcopy each evaluate complementary manifestations of *S. haematobium* infection, LCA offers an appropriate framework for integrating information from all available tests without assuming that any single method is perfectly accurate.

In this study, we compared the diagnostic performance of genital swab real-time PCR, urine microscopy and colposcopy for the detection of FGS among women living in *S. haematobium*-endemic communities in Cameroon. A two-class latent class model was used to estimate the sensitivity and specificity of each diagnostic method and infer the underlying infection status in the absence of a diagnostic gold standard. By combining molecular, parasitological and clinical diagnostic approaches within a latent class framework, this study provides a comprehensive evaluation of FGS diagnostics and contributes evidence to support improved surveillance and disease management in endemic settings.

## Methods

### Ethics statement

Ethical approval (Ref N° 2022/08/1485/CE/CNERSH/SP) was obtained from the Cameroon National Ethics Committee on Human Health Research. Administrative authorizations were gotten from the Regional Delegation of Public Health for the West Region of Cameroon (Ref N˚ 281/L/MINSANTE/SG/DRSPO/CBF), the District Medical Officer for the Malanteoun Health District and the Magba Sub-Divisional Office. In accordance with the Declaration of Helsinki, signed informed consent was obtained from all female participants. Women aged 18 years and above who resided in the study communities, provided written informed consent, and were willing to provide urine and cervical swab samples and undergo pelvic examination were eligible for inclusion. Sexually active female participants, less than 18 years gave assent while their parents, husbands, or guardian gave consent for them to participate. Also verbal consent was obtained from husbands or partners of the women before they were approached as required by the cultural regulations. Participants were informed of the purpose of the study, the risks associated with the sample collection methods, and the potential benefits to public health. Interviews and questionnaires were administered in their local languages. Positive cases were referred to the Chief of Centre of health facility for point of care and treatment. Participants were excluded if they were pregnant, had undergone hysterectomy, had a known history of cervical cancer or treatment for cervical lesions, were receiving treatment for schistosomiasis within the preceding six months, or declined any of the study procedures.

### Study site and design

**Fig. 1:**
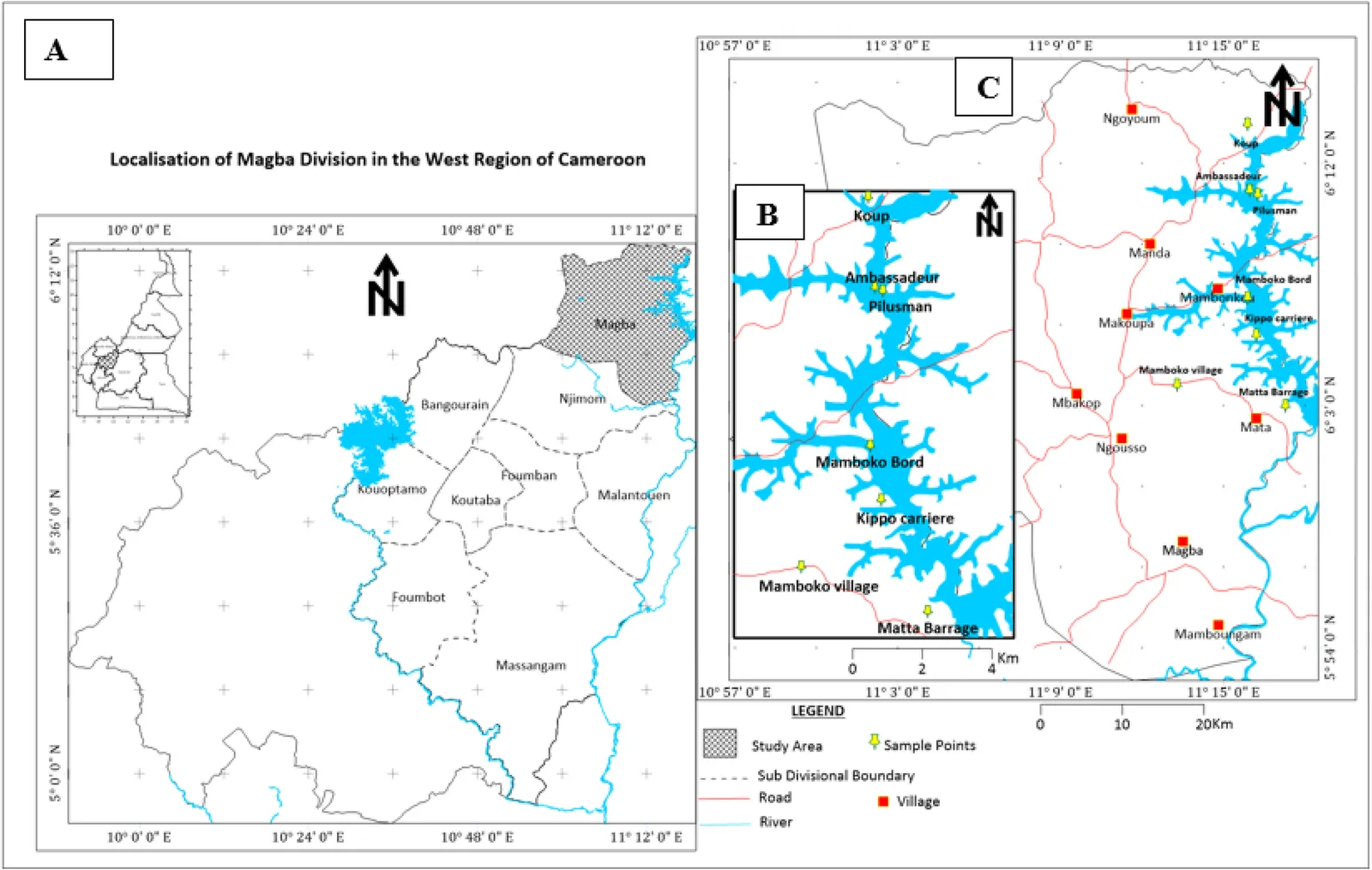
Map showing **A**: Magba division. **B**: Mape Dam and communities in the Matta Health area and **C**: sampled communities in the West Region of Cameroon

This study was carried out on the islands of Matta barrage under the Matta health area. The Matta Health Area is one of the health areas under the Malanteoun Health District in the West Region of Cameroon. For decades now, the Malanteoun health district has been known to house hotspot areas with high transmission after treatment for schistosomiasis in Cameroon, amongst which Matta health area is the most significant (Makia et al. 2023). Six communities (Kippo-Carrier, Mambonkor village, Ambassadeur, Mambonkor Bord, Koup, Matta-barrage and Pilinusman1) (Fig. 1) were screened due to the presence of a water barrage constructed in the early 1960s as a diversion for the Mape Dam (3,300 million m 3). The islands are only accessible by boats only meanwhile, the mainland are accessible by road and both communities practice mainly fishing and farming as their routine activities. Over the years, settlers migrated from different local ethnic groups mostly from the Far North (Massa, Kotoko, Mosgum, Arab), North West and West (mostly the Tikars, who are the indigenes of this area) regions of Cameroon, and different countries (Mali and Nigeria) to practice fishing for business purposes.

### Recruitment strategy, study period and sample size

Mobilization and sensitization activities preceded the diagnostic screening. The study period spanned across the months of mid-July to -August 2024. Participants were contacted within the community through the community drug distributors or health workers. They distributed flyers while at the same time explained the different diseases prevalent in the community. While local primary healthcare facilities were present in the study area, clinical screenings were conducted within private, designated spaces at traditional community leaders’ compounds. This strategy was deployed to respect local cultural protocols, reduce geographical access barriers for rural participants, and maximize patient privacy for a highly stigmatized condition.

No study has been carried out in this area to determine the prevalence of *Schistosoma* DNA using swabs. So, we made use of the prevalence (18.1%) reported by (Randrianasolo et al. 2015) using vaginal lavage samples.

For an objective that estimates a prevalence, the sample size calculation was done using the following formula by (Naing et al. 2022):

N = Z _α/2_^2^ p (1-p) / MOE2, where Z_α/2_ is the critical value of the Normal distribution at α/ 2 (for the confidence level of 95%, α is 0.05 and the critical value is 1.96), MOE is the margin of error (5%), p is the prevalence (0.181). Our sample size with expected specificity and sensitivity of 90% was 192.

### Sample and data collection

Each participant was questioned in a private room on their reproductive life experiences, signs and symptoms. Also, the demographic data of each participant was collected. After this, screw capped urine containers were provided carrying their identification information to provide urine at least three times in a day. About 60 ml urine was collected from each participant. The samples were analyzed immediately for microhaematuria and proteinuria using urine reagent strips (URS 11, Cortez Diagnostics, INC, USA). Urine-egg microscopy was used to diagnose urogenital schistosomiasis and quantify expelled *S. haematobium* ova according to a standard protocol (Cheesbrough 1985). The urine samples were processed using the membrane filtration technique and examined microscopically for the presence of *S. haematobium* infection based on morphology of the ova. The urine sample was gently agitated by hand and 10 ml of urine was drawn with a syringe, filtered through membrane filter (Sterlitech Polycarbonate (PCTE) membrane filters, USA), the filter was removed, placed on a glass slide using a forcep and stained with 1% Lugol’s iodine solution (Cheesbrough 1985). The slide was then examined using the Binocular Compound light microscope. Terminal-spined eggs, characteristics of *S. haematobium* were identified and counted manually. Three urine samples were collected from each participant during the same day and pooled. A 10 mL aliquot of the pooled urine was subsequently filtered and examined microscopically for *S. haematobium* eggs. Positive urine-egg microscopy preparations were scored according to the number of *S. haematobium* eggs observed per 10 mL urine using the following categories: light-moderate infection (1–49 ova per 10 mL urine); heavy infection (≥ 50 ova per 10 mL urine) (WHO 2002). To ensure rigorous quality control for egg detection, all processed urine filtration slides were read by a primary technician. Subsequently, a random 10% subset of negative slides and 100% of positive slides were independently re-examined by a senior microscopist blinded to the initial outcomes, with any diagnostic discrepancies resolved by joint consensus.

### Pelvic examinations and sampling of females for FGS

Simultaneous examination of the females by the gyneacologist (OKS) was done alongside the detection of *S. haematobium* in urine. Patients were interviewed in their local language. Pelvic examination was done in the following sequence: swab collection and immersed in DNA/RNA shield, and stored at − 20 °C and Colposcopic images (9800 A Digital video colposcope with camera, China, magnifications). Homogenous yellow sandy patches were defined as sandy looking areas with no distinct grains using 15-times magnification. Grainy sandy patches constituted grains (~ 0.05 by 0.2 mm) situated in the mucosa. Neovascularization was defined as pathological convoluted (cork-screw), reticular, circular and/or branched, uneven-caliber blood vessels visible (by 15 times magnification) on the mucosal surface. Female genital schistosomiasis (FGS) was defined as the presence of sandy patches, abnormal blood vessels, and rubbery papules in genital tissue. Contact bleeding was defined as fresh blood originating from the mucosal surface. Pre-contact bleeding was defined as darkened blood on the mucosal surface in the absence of recent or present menstruation (WHO 2015; Sturt et al. 2023). A “positive case” for treatment referral was defined as (presence of characteristic FGS lesions on visual inspection and/or laboratory confirmation by microscopy). Participants meeting this criterion were referred for treatment according to national schistosomiasis management guidelines. The gyneacologist did the primary diagnosis and noted the results, two researchers AN and GBW who had been trained to diagnose FGS conducted a blind assessment of the pictures and the results were compared. In cases of disagreement, images were jointly reviewed and discussed to reach a consensus.

## Molecular analysis

### DNA extraction

DNA was extracted directly from genital swab samples which were preserved in DNA/RNA shield using a commercial genomic DNA extraction kit, Zymo DNA miniprep kit (Zymo Research, USA) according to the manufacturer’s instructions. 20ul of Proteinase K and 200 ul Biofluid/cell Buffer was added to 200ul of the sample/shield mixture. It was thereafter mixed thoroughly /vortexed for 10–15 s and then incubated at 55 °C for 10 min. This was then followed by adding 420ul of genomic binding buffer to the digested sample and vortexed for 10–15 s. The mixture was then transferred to a Zymo-spin IIC-XLR column in a collection tube and centrifuged at ≽12,000 g for 1 min. After three wash/centrifugation steps, the spin column was thereafter transferred into a clean micro centrifuge tube, and 50ul DNA elution Buffer added directly on the matrix. This was incubated for 5 min at room temperature then centrifuged at maximum speed for 1 min to elute the DNA and stored at ≤ -20 °C.

## Quantitative-PCR by SYBR green (melt curve analysis) and probe-based assays

Real-time qPCR was used to detect *S. haematobium* in genital swabs by targeting the *Dra1* sequence. The highly repetitive *Dra1* sequence of *S. haematobium* (Accession number DQ157698.1) was selected based on a previous study described by (Hamburger et al., 1991) (Sh-FW *5-gatctcacctatcagacgaaac-3′*; Sh-RV *5′-tcacaacgatacgaccaac-3′*). SYBR green for real-time monitoring of the PCR signal was used and a melt curve was generated to confirm the specificity of the amplicons generated. A 20 µL total reaction mix volume with 5 µL DNA, 10nM of Sh-FW and Sh-RV primer. Amplification consisted of an initial step of 60 s at 95°C followed by 45 cycles of 15 s at 95°C and 60 s at 60°C. The reaction was run on the CFX opus (Bio-rad, USA). Melt curve was run for 1 cycle at 60–95°C. DNA detection was expressed by Cycle threshold (Ct)-values and confirmed by melt curve analysis (Supplementary Fig. 1). To ensure the robustness of our PCR results, additional confirmatory analysis were conducted using Probe based assays (Probe 5’-6FAM TGTTGGTGGAAGTGCCTGTTTCGCAA-MGB- 3’) and a size separation of the different amplicons was carried out to identify the 121 bp *S. haematobium* amplicon alongside a molecular weight ladder to confirm the specificity of all the PCR amplicons generated (Supplementary Fig. 2). The reaction was run according to the Luna Universal Probe qPCR master mix protocol (New England Biolabs, M3004S).

### Control samples

Nuclease free water was used as a no template control while a pool of *S. haematobium* eggs DNA obtained from the filtered urine was used as a positive control. For the Probe assay, the *S. haematobium* target sequence was synthesized with a known concentration and used as standard.

#### **Statistical analysis**

All analyses were conducted on a sample of *N* = 143 participants. Epidemiological data were entered, cleaned and validated in a Microsoft™ excel spreadsheet (MS Office Excel^®^ 2016). Schistosomiasis test results: PCR, eggs in urine and colposcopic descriptions (positive or negative) were the dependent variable. The data were then exported to SPSS software ver. 21 (IBM Corp., Armonk, NY, USA). A univariate analysis was conducted for descriptive results such as frequencies and proportions. An analysis of association with *Schistosoma* DNA, FGS and egg counts positivity was conducted using Pearson chi-square (and Fisher’s exact test where appropriate) in a bivariate analysis. For the multivariate analysis, a stepwise logistic regression method algorithm was used, then independent variables (p≺0.05) were considered significant risk factors of FGS. “Inter-observer agreement between expert reviewers for the presence of female genital schistosomiasis (FGS) was assessed using Cohen’s kappa (κ) statistic. Agreement was interpreted according to Landis and Koch criteria as poor (< 0.00), slight (0.00–0.20), fair (0.21–0.40), moderate (0.41–0.60), substantial (0.61–0.80), or almost perfect (> 0.80) (Landis and Koch 1977). Agreement between visual FGS diagnosis and laboratory methods (PCR and microscopy) was similarly evaluated using Cohen’s kappa. Associations between diagnostic methods were assessed using chi-square test. Statistical significance was set at *p* < 0.05.

#### Latent class analysis

LCA incorporating PCR, colposcopy, and urine egg microscopy was conducted to estimate the sensitivities and specificities of each diagnostic test without assuming any test to be perfect (Keddie et al. 2023). LCA was implemented in RStudio version 4.2.1 using a custom EM algorithm. The results of colposcopy, microscopy and real-time PCR tests were combined as indicators of an underlying latent class. The latent variable was defined as the unobserved FGS status (FGS present vs. FGS absent). Urine microscopy, genital PCR and colposcopy were included as complementary indicators of this latent construct. Although urine microscopy primarily detects active urinary egg excretion rather than genital pathology, it was incorporated because FGS occurs as a consequence of *S. haematobium* infection, and active egg excretion provides supportive evidence of the underlying infection from which genital disease arises. .

Because no single diagnostic method evaluates Female Genital Schistosomiasis (FGS) with perfect accuracy, a two-class Latent Class Analysis (LCA) was executed to estimate the disease prevalence and evaluate the diagnostic accuracy of each test. LCA is a mathematical profiling technique that treats the infection status as an unobserved (latent) categorical variable with two distinct classes: C = 1 (uninfected) and C = 2 (infected). The model combines the joint conditional probabilities of the three observed binary indicators: Polymerase Chain Reaction (PCR), Urine Egg Microscopy, and Colposcopy/Visual FGS.

The likelihood function of the LC model for a random sample of N individuals is (Ibironke et al. 2012)

$$\;\begin{array}{c}\:L\left(X\right)=\prod_{i=1}\limits^{N}\\\left(\eta\prod_{j=1}\limits^{d}{\pi\:}_{j1}^{xij}{\left(1-{\pi\:}_{j1}\right)}^{1-xij}+\left(1-\eta\right)\prod_{j=1}\limits^{d}{\pi\:}_{j0}^{xij}{\left(1-{\pi\:}_{j0}\right)}^{1-xij}\right)\end{array}$$

Where L(X) represents the joint likelihood of the observed diagnostic data across the sample population (*N* = 143), and (d) represents the number of distinct diagnostic modalities (d = 3; PCR, Urine Egg Microscopy, and Colposcopy/Visual FGS). The variable ϰij denotes the binary indicator outcome (1 for positive and 0 for negative) for individual *i* on diagnostic test *ϳ*. The parameter Ƞ represents the latent class prevalence of the infected cohort (Class 2), while (1- Ƞ) represents the prevalence of the uninfected cohort (Class 1). The conditional probability $\:{\pi\:}_{j1}$ signifies the sensitivity profile of test *j* within the infected class, and$\:{\:\pi\:}_{j0}$ denotes the false-positive probability of test *j* within the uninfected class, with $\:(1-{\pi\:}_{j0})$ defining the specificity parameter:

The model relies on the assumption of local independence. This dictates that within any single latent class, the execution and results of the three diagnostic tests are statistically independent of one another. Maximum likelihood parameters were generated iteratively using the Expectation-Maximization (EM) algorithm via the poLCA package in R.

Latent classes were clinically identified based on their item-response profiles. Class 2 was definitively established as the “infected cohort” due to its 100% conditional probability for positive PCR outcomes. Diagnostic sensitivities were extracted from the conditional probabilities of a positive test (1) given membership in Class 2 represented as $\:{\pi\:}_{j1}$. Specificities were extracted from the conditional probabilities of a negative test (0) given membership in Class 1 computed as $\:(1-{\pi\:}_{j0})$.

To account for precision errors in small class sample divisions, 95% Confidence Intervals (CI) for these diagnostic metrics were calculated via Wald sample proportion intervals based on the estimated class-specific denominators (n_**infected**_ = 130; n_**healthy**_= 13).        

## Results

### Prevalence of schistosomiasis in the urine and cervix

The overall prevalence of urinary schistosomiasis in the study population was 29.37%, with a egg count range of 2-540 eggs per 10mL of urine. Furthermore, cervical schistosomiasis (positive *S. haematobium* DNA), was observed in 93% of the women examined by SYBR green PCR and 73.43% by the probe based PCR (Table 1).

**Table 1:** Prevalence of urogenital schistosomiasis and *Schistosoma* DNA in the cervix

RT-PCR values	Egg groups (number of eggs/10 ml urine)					
	0	1–49	≽50	Total	Disease prevalence in cervicovaginal swab sample	*P*-value
SYBR green					93%	0.67
Positive (Ct≺28, Strong)	18	10	5	33		
Positive (Ct:28–38, moderate)	75	19	6	100		
Negative (Ct:≻38 or 0 weak)	8	2	0	10	7%	
Total	101	31	11	143	100%	
Probe based + Agarose						
Positive	73	25	7	105	73.43%	0.63
Negative	28	6	4	38	27.27%	
Total	101	31	11	143	100%	
Visual FGS						
Positive	19	11	4	34	23.7%	0.04*
Negative	81	20	8	109	76.2%	
Total	102	30	12	143	100%	
Prevalence of schistosomiasis egg excretion	70.6% (Negative)	29.4% (Positive)		100%	-	

Ct-values indicate a strong (Ct, 28), medium (Ct between 28–38), weak (Ct ≻38) or no (Ct = 0) recognition of the species (Cnops et al. 2013)

The relationship between urine egg microscopy infection status (negative and positive) and the positivity of the diagnostic tests was assessed using the chi-square test. No significant difference was observed between PCR positivity and microscopy infection status categories for either the SYBR-based assay (χ² = 0.4551, *p* = 0.67) or the probe-based assay (χ² = 0.2328, *p* = 0.63). In contrast, the frequency of colposcopic findings suggestive of Female Genital Schistosomiasis differed significantly according to microscopy status (χ² = 4.186, *p* = 0.04), with a higher proportion of visual FGS lesions among participants with detectable urinary egg excretion than among microscopy-negative individuals. (Table 1).

There was substantial agreement between expert reviewers for the diagnosis of FGS based on visual inspection with a concordant diagnosis in 90.2% (129/143) participants (33 positive and 96 negative) and discordant diagnosis in 9.8% (14/143) participants. The inter-observer agreement yielded a Cohen’s kappa value of κ = [0.76] (95% CI: [0.84–0.94]), indicating substantial concordance between reviewers. Cohen’s kappa is restricted to those participants where both Reviewers provided diagnosis. No significant associations were observed with the visual FGS diagnosis and PCR FGS /eggs in urine (Table 2).

**Table 2:** Agreement of expert reviewers for the presence of FGS and associations of the visual FGS diagnostics with PCR and microscopy

Reviewer 1						
Schistosomiasis diagnosis		Visual FGS not detected (77%)	Visual FGS detected (23%)	Crude OR (95% CI)	*P*-value	Cohen’s Kappa (CI] (Interpretation)
Eggs in urine	Detected	28(19.58)	14(9.79)	0.46 (0.21–1.04)	0.17	0.76(0.84–0.94) (Substantial agreement)
	Not detected	82(57.3)	19(13.29)	Ref		
PCR	Detected	83(58)	22(15.4)	1.54(0.66– 3.58)	0.32	
	Not detected	27(18.9)	11(7.7)	Ref		
Reviewer 2						
Schistosomiasis diagnosis		Visual FGS not detected (67.1%)	Visual FGS Detected (32.9%)	Crude OR (95% CI)	P-value	
Eggs in urine	Detected	25(17.5)	17(11.9)	0.62(0.29–1.32)	0.48	
	Not detected	71(49.7)	30(21)	Ref		
PCR	Detected	70(48.95)	35(24.48)	0.92(0.42–2.05)	0.84	
	Not detected	26(18.2)	12(8.39)	Ref		

### Association between *S. haematobium* prevalence and sociodemographic characteristics

The study population comprised 143 women, predominantly originating from the Adamawa, Far North, Northwest, and West regions of Cameroon, as well as Benue State in Nigeria, who had settled on the islands in Cameroon. Over 47.6% of participants had resided in their respective regions for 10 or more years. The mean age of participants was 23.39 ± 4.28 years [15–24 (37.1%); 25–34 (35.7%); 35–44 (19.8%) and ≻45 (7.69%)]. Just over half of the participants (53.2%) identified their primary occupation as housewife meanwhile, profession, region of origin and community were significantly associated with egg count. Community and type of marriage were significantly associated with the total number of FGS signs (Table 3). 

**Table 3:** Associations between demographic characteristics of participants, *S. haematobium* DNA in cervix, FGS signs and egg count

Demographic characteristics	Number of respondents *N* (%)	Sh DNA positive *N* (%)	Sh DNA positive OR (95% CI)	Egg positive *N* (%)	Egg positive OR (95% CI)	Reviewer 1 FGS + VE *N* (%)	Reviewer 2 FGS + VE *N* (%)	FGS positive OR (95% CI)
Age		*p* = 0.327		*p* = 0.17		*P* = 0.219		
• 15–24	53 (37.1)	40 (27.97)	0.39 (0.27–0.56)	21 (14.69)	0.17(0.11–0.27)	11 (7.69)	14(9.79)	0.1(0.05–0.17)
• 25–34	51 (35.7)	39 (27.27)	0.38 (0.26–0.54)	13 (9.1)	0.1(0.06–0.18)	14(9.79)	19(13.29)	0.13(0.08–0.22)
• 35–44	28 (19.58)	20 (13.99)	0.16(0.10–0.26)	7 (4.89)	0.05(0.02–0.11)	9 (6.29)	11(7.69)	0.08(0.04–0.14)
• ≻45	11(7.69)	6 (4.2)	0.04 (0.02–0.1)	1(0.69)	0.007(0.001–0.04)	3 (2.1)	5(3.5)	0.03(0.01–0.07)
Profession		*p* = 0.126		*p* = 0.001^*^		*P* = 0.12		
• Fisher woman	24 (16.8)	14 (9.79)	0.12 (0.06–0.19)	16 (11.18)	0.13 (0.07–0.17)	10 (6.99)	11(7.69)	0.08(0.04–0.15)
• Farmer	33 (23.1)	24 (16.78)	0.20 (0.13–0.31)	7 (4.89)	0.05 (0.02–0.11)	8 (5.59)	12(8.39)	0.08(0.04–0.14)
• Housewife	76 (53.2)	60 (41.96)	0.72 (0.52–1.10)	19 (13.29)	0.15 (0.1–0.25)	18(12.59)	24(16.78)	0.17(0.11–0.27)
• Student	4 (2.8)	1 (0.70)	0.007(0.001–0.04)	0 (0)	-	1 (0.70)	1(0.70)	0.007(0.001–0.04)
• Trader	6 (4.2)	6 (4.2)	0.044 (0.02–0.1)	0 (0)	-	0	1(0.69)	-
Region of origin		*p* = 0.86		*p* = 0.001^*^		*P* = 0.08		
• Adamawa region	12(8.4)	10 (6.99)	0.08(0.04–0.14)	4 (2.8)	0.03(0.01–0.07)	1(0.70)	3(2.1)	0.01(0.004–0.05)
• Extreme north	41(28.7)	30 (20.98)	0.27(0.18–0.40)	10 (6.99)	0.08(0.04–0.14)	6 (4.2)	10(6.99)	0.06(0.03–0.12)
• Nigeria-Benue	35(24.5)	19 (13.29)	0.15 (0.1–0.25)	19 (13.29)	0.15(0.1–0.25)	13 (9.09)	15(10.49)	0.11(0.06–0.19)
• North west region	3 (2.1)	3 (2.1)	0.02 (0.007–0.06)	2 (1.4)	0.01(0.004–0.05)	2 (1.4)	3(2.01)	0.01(0.004-0.1)
• West region	52(36.4)	43 (30.07)	0.43(0.30–0.61)	7 (4.9)	0.05 (0.02–0.11)	13 (9.09)	16(11.19)	0.11(0.07–0.19)
Community		*p* = 0.218		*p* = 0.001^*^		*P* = 0.03*		
• Ambassadeur	1(0.7)	0(0)	-	0 (0)	-	0	0	-
• Kippo-Carrier	19 (13.3)	16 (11.19)	0.13(0.08–0.21)	8 (5.59)	0.06(0.03–0.11)	9(6.29)	8 (5.59)	0.14 (0.08–0.22)
• Koup	18 (12.6)	11 (7.69)	0.08(0.05–0.15)	7 (4.9)	0.05 (0.02–0.11)	5(3.5)	7(4.9)	0.044 (0.02–0.1)
• Mambonko village	35 (24.5)	28 (19.58)	0.24(0.16–0.37)	3 (2.1)	0.02 (0.007–0.06)	9(6.29)	12(8.39)	0.08(0.04–0.15)
• Mambonkor bord	27 (18.9)	24(16.78)	0.20(0.13–0.31)	10 (6.99)	0.08(0.04–0.14)	2 (1.4)	6 (4.2)	0.06(0.03–0.11)
• Matta – barrage	22 (15.4)	16 (11.19)	0.13(0.08–0.21)	2 (1.4)	0.01(0.004–0.05)	4 (2.8)	7(4.9)	0.04(0.02–0.09)
• Pilinusman 1	21 (14.7)	10(6.99)	0.08(0.04–0.14)	12 (8.39)	0.09(0.05–0.16)	8 (5.59)	9(6.29)	0.14 (0.08–0.22)
Number of years in village		*p* = 0.753		*p* = 0.790		*P* = 0.39		
• 0–1 year	7(4.9)	6(4.2)	0.12(0.06–0.19)	4 (2.8)	0.03(0.01–0.07)	2(1.4)	2 (1.4)	0.01(0.004–0.05)
• 2–10 years	65(45.45)	47(42.87)	0.49(0.35–0.69)	20 (13.99)	0.16(0.10–0.26)	13(9.1)	17 (11.89)	0.12(0.07–0.2)
• ≻10 years	68(47.6)	48 (33.57)	0.50(0.36–0.71)	18 (12.59)	0.14(0.09–0.23)	20(13.99)	28 (19.58)	0.2(0.13–0.31)
Educational status		*p* = 0.568		*p* = 0.463		*P* = 0.203		
• Secondary/high school	16 (11.2)	14 (9.79)	0.11(0.06–0.19)	3 (2.09)	0.02 (0.007–0.06)	4(2.8)	4(2.8)	0.02(0.01–0.07)
• Primary	49 (34.3)	36 (25.2)	0.34(0.23–0.49)	12 (8.39)	0.09(0.05–0.16)	16(15.38)	20(15.38)	0.13(0.07–0.21)
• Analphabet	78(54.5)	55 (38.46)	0.63(0.45–0.87)	27 (18.88)	0.23(0.15–0.35)	17(11.89)	25(17.48)	0.17(0.11–0.27)
Marital status		*p* = 0.061		*p* = 0.199		*P* = 0.65		
• Divorce /widow	4 (2.8)	4 (2.8)	0.03 (0.01–0.07)	0 (0)	-	1(0.7)	2(1.4)	0.01(0.004–0.05)
• Married	125 (87.4)	94 (65.73)	1.91 (1.36–2.71)	35 (24.48)	0.32(0.22–0.47)	32(22.38)	43(30.07)	0.36(0.25–0.51)
• Single	14 (9.8)	7 (4.9)	0.05 (0.02–0.11)	7 (4.9)	0.05 (0.02–0.11)	4(2.8)	4(2.8)	0.03 (0.01–0.07)
Type of marriage		*p* = 0.05^*^		*p* = 0.626		*P* = 0.04*		
• Monogamy	78(54.5)	58 (40.56)	0.68 (0.49–0.95)	24 (16.78)	0.2(0.13–0.31)	23(16.08)	27(18.9)	0.21(0.14–0.33)
• Polygamy	47(32.9)	35 (24.5)	0.32(0.22–0.47)	12 (8.39)	0.09(0.05–0.16)	9(6.29)	15(10.49)	0.09(0.05–0.16)
• Single	6(4.2)	2(1.4)	0.01(0.004–0.05)	3 (2.1)	0.02 (0.007–0.06)	2(1.4)	2 (1.4)	0.01(0.004–0.05)
• Not applicable	12(8.4)	10(6.99)	0.08(0.04–0.14)	3 (2.1)	0.02 (0.007–0.06)	3(3.50)	5(4.2)	0.03 (0.01–0.07)

Statistical significance was considered at *p≺0.05

The potential risk factors when exposed to *Schistosoma* parasite leading to egg excretion in urine identified in our study were lack of treatment (OR: 0.35 95%CI: 0.13–0.98) and fishing (OR: 3.37 95% CI: 1.45–7.8) (Table 4).

**Table 4:** Potential risk factors for the participants

Variable	Egg counts OR (95%CI)	*P*-value
Age		
• ≻45	1	
• 15–24	0.201(0.023–1.749)	0.146
• 25–34	0.411(0.046–3.684)	0.427
• 35–44	0.371 (0.039–3.544)	0.389
Years lived in the village		
• ≻10 years	1	
• 0–1 year	1.42 (0.358–5.652)	0.616
• 2 to 10 years	1.14 (0.48–2.71)	0.769
Laundry		
• Yes	1.9 (0.52–6.9)	0.33
• No	1	
Fishing		
• Yes	3.37 (1.45–7.8)	0.005*
• No	1	
Lack of treatment		
• Yes	0.35 (0.13–0.98)	0.05*
• No	1	

Odds ratios were adjusted using Bonferroni correction (*p≺0.05 statistically significant)

### Prevalence of urogenital and gynecological signs and symptoms

**Fig. 2:**
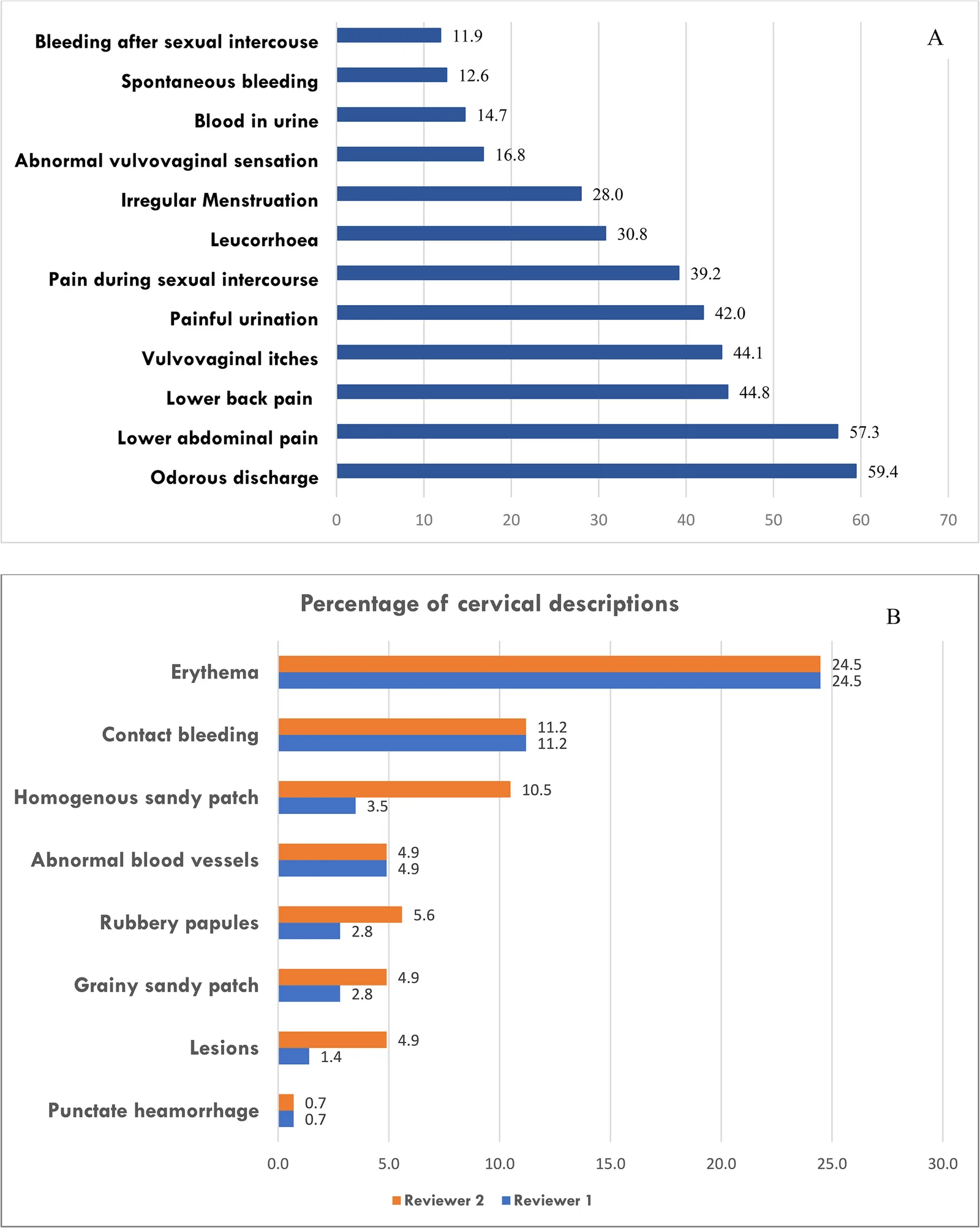
**a**) Percentage distribution of symptoms and urogenital complaints **b**) distribution of cervical abnormalities in the study population and reported signs/symptoms in the study population (N=143)

A total of 138/143 participants reported at least one of the urogenital signs and symptoms making an overall prevalence of 96.5%. The most reported signs and symptoms included: lower abdominal pain (57.3%), vulvovaginal itches (44.1), odorous discharges (59.4%), lower back pain (44.8%), painful micturition (42%) and pain during sexual intercourse (39.2%) (Fig. 2a). Also, the most observed cervical abnormality reported by reviewers was erythema, contact bleeding, homogenous sandy patches, rubbery papules, lesions and grainy sandy patch (Fig. 2b). Worth noting, 15/21(71.4%) participants with erythema only signs were PCR positive for *S. haematobium* and 3/21 (14.3%) egg positive. 

### Characterization of FGS signs, pelvic symptoms and associations with PCR and egg count

Specific to FGS, 33/143 (23.08%) participants had at least one typical FGS signs and symptoms. Intermediate non-specific cases for FGS were those having contact bleeding, erythema and puntate hermorhages signs similar to other sexually transmitted infections. Among those with FGS, 9% (13/143) had single FGS symptoms, while 14% (20/143) had multiple symptoms. While 18 different multiple FGS signs and symptoms combinations were theoretically possible, we observed distinct symptoms combinations: (i) Contact bleeding, erythema, rubbery papules (ii) Contact bleeding, Homogenous Sandy patches (iii) Homogenous Sandy patches, contact bleeding, abnormal blood vessels (iv) Erythema, rubbery papules at 4 & 5 Oclock (v) Abnormal blood vessels, rubbery papules. Homogenous sandy patch, blood in urine, joint pains, painful urination and vulvovaginal itches were the signs closely associated with *Schistosoma* egg excretion (Table 5).

**Table 5:** Associations between PCR, egg excretion and pelvic signs and symptoms

	Cervicovaginal S. haematobium DNA			Urinary S. haematobium egg count		
	Positive (*n* = 105)	Negative (*n* = 38)	*P*-value	Positive (*n* = 40)	Negative (*n* = 103)	*P*-value
Urogenital complaints (*N* = 138/143)						
Lower back pain (64)	49	15	0.49	20	44	0.43
Vulvovaginal itches (63)	58	22	0.81	29	51	0.014*
Painful urination (60)	44	16	0.97	23	37	0.018*
Joint pains (37)	31	6	0.075	14	80	< 0.0001*
Leucorrhoea (44)	34	10	0.49	15	29	0.28
Irregular menstruation (40)	29	10	0.94	14	25	0.27
Odorous discharge (85)	68	10	0.036	21	64	0.37
Lower abdominal pain (82)	65	17	0.35	23	59	0.98
Pain during sexual intercourse (56)	41	15	0.98	17	39	0.67
Bleeding after sexual intercourse (17)	14	3	0.66	4	13	0.71
Abnormal vulvovaginal sensation (24)	19	5	0.72	7	17	0.90
Spontaneous bleeding (18)	17	1	0.27	8	10	0.16
Blood in urine (21)	17	4	0.62	12	28	0.04*
Cervical description (*N* = 52/143)						
Erythema (35)	21	14	0.27	9	26	0.77
Contact bleeding (16)	9	7	0.40	3	13	0.46
Lesions (6)	5	1	0.77	1	5	0.11
Abnormal blood vessels (7)	7	0	0.40	1	6	0.48
Rubbery papules (7)	3	2	0.72	2	3	0.66
Grainy sandy patches (4)	4	0	0.81	4	0	0.43
Homogenous sandy patch (16)	13	3	0.68	9	7	0.03*
Punctate herrmorages (1)	1	0	0.964	1	0	0.294

*Statistical significance (0.0001 ≤ p≺0.05)

### Diagnostic performance of the assays

The apparent positivity rates differed across the three diagnostic methods, with PCR yielding the highest positivity rate of 73.4%, followed by urine egg microscopy (29.4%) and colposcopy (23.08%). Response pattern analysis showed considerable diagnostic variance between methods, particularly among women who were PCR-positive but negative by both urine microscopy and colposcopy.

### Latent class selection

A two-class Latent Class Analysis (LCA) model was fitted to evaluate the diagnostic data of 143 participants across three modalities: PCR, Urine Egg Microscopy, and Colposcopy/Visual FGS. The model successfully converged using the Expectation-Maximization (EM) algorithm, resolving the sample population into two distinct clusters based on test item-response profiles. Latent class analysis generated a model-estimated prevalence that exceeded the apparent prevalence obtained using each individual diagnostic test. The apparent prevalence was 73.43% for cervical Dra1 probe-based PCR, 30.77% for urine egg microscopy, and 22.38% for colposcopy. These findings suggest that reliance on a single diagnostic method may underestimate the occurrence of FGS.

### Diagnostic performance and conditional item probabilities

The conditional item-response probabilities generated by the LCA model revealed a stark divergence in performance between molecular testing and conventional diagnostic methods. Latent Class 2 was definitively identified as the infected cohort due to a perfect conditional probability of a positive PCR result ($\:{\pi\:}_{PCR,1}=1.000$). Conversely, Class 1 represented the uninfected cohort, demonstrating a 95.20% conditional probability of a negative PCR outcome ($\:1-{\pi\:}_{PCR,0}=0.9520$). This established a PCR diagnostic sensitivity of 100.00% (95% CI: 94.85% – 100.00%) and a specificity of 95.20% (95% CI: 91.88%– 98.52%). In contrast to PCR, both conventional diagnostics exhibited high false-negative rates within the infected cohort. For Urine Egg Microscopy, the probability of returning a positive result in the infected class ($\:{\pi\:}_{micro,1}$) was only 0.2906, yielding an operational sensitivity of 29.06% (95% CI: 17.5% – 40.62%). Colposcopy/Visual FGS performed similarly, with a conditional positive probability ($\:{\pi\:}_{colpo,1}$) of 0.3827, resulting in a sensitivity of 38.27% (95% CI: 29.92% – 46.62%).

However, both traditional methods demonstrated strong rule-out capabilities within the healthy cohort (Class 1). The conditional probability of testing negative given a healthy state was 0.8332 for Urine Egg Microscopy ($\:1-{\pi\:}_{micro,0}$) and 0.8333 for Colposcopy/Visual FGS ($\:1-{\pi\:}_{colpo,0}$). This translates to a diagnostic specificity of 83.32% (95% CI: 62.75% – 100.00%) for microscopy and 83.33% (95% CI: 62.76% – 100.00%) for colposcopy.

The complete distribution of the model parameters, along with their corresponding 95% confidence intervals capped within logical diagnostic boundaries, is detailed in Table 6.

**Table 6:** Diagnostic performance of the various diagnostic methods using latent class analysis

Diagnostic test	Sensitivity % (95% CI)	Specificity % (95% CI)
PCR	100.00% (94.85–100.00)	95.20% (91.88–98.52)
Urine egg microscopy	29.06% (17.5–40.62)	83.32% (62.75–100.00)
Colposcopy / Visual FGS	38.27% (29.92–46.62)	83.33% (62.76–100.00)

## Discussion

Reliable diagnosis is fundamental to the effective management and control of FGS, particularly in endemic settings where no definitive diagnostic gold standard exists. This study therefore evaluated the complementary performance of urine egg microscopy, cervical Dra1 probe-based PCR, and colposcopy using latent class analysis among women from six endemic communities in the Matta Health Area of the West Region of Cameroon.

Differences were observed in the diagnostic performance and case detection profiles of urine egg microscopy, colposcopy, and cervical PCR. PCR identified a higher proportion of women with detectable *S. haematobium* DNA than urine egg microscopy or colposcopy, and latent class analysis similarly estimated a higher sensitivity for PCR than for the other diagnostic methods. These findings are consistent with the different biological targets of the tests: PCR detects parasite DNA in the genital tract, urine microscopy identifies active urinary egg excretion, and colposcopy detects characteristic genital lesions associated with FGS. Consequently, each method captures different manifestations of infection and disease. The higher PCR positivity may therefore reflect the detection of parasite DNA in women with low-intensity or intermittent infections, localized genital involvement, or persistent DNA following recent or past infection (Baltrušis and Höglund 2023). However, because PCR does not distinguish between active infection, persistent parasite DNA, or recent exposure, molecular results should be interpreted alongside parasitological and clinical findings. Together, these observations support the complementary use of molecular, parasitological, and clinical approaches for the evaluation of FGS in endemic settings.

A plausible reason for high *Schistosoma* DNA in cervical samples could be due to continuous exposure and dependency of the study participants to the water dam surrounding their islands leading to sustained active infection. Moreover, cervical lesions observed are a confirmation to the long-term chronicity of infection due to many years of exposure to *S. haematobium* infection. The lower median Ct value observed among women with colposcopically evident FGS suggests a higher burden of *S. haematobium* DNA in the genital tract compared with women without visual FGS. Although PCR positivity alone cannot distinguish active infection from persistent parasite DNA, these findings support the complementary use of molecular and colposcopic assessment in the evaluation of FGS. The number of eggs and FGS signs decreased with age reflecting concomitant partial immune response to schistosomiasis new infections (Clegg et al. 1971; Agnew et al. 1996). It has been suggested that the presence of host antigens in chronic infections serves to mask the parasite antigens at the surface of the older stages and thus prevents rejection by the immune response of the host. Also, young schistosomula do not possess these masking antigens and are therefore vulnerable to the immune reaction which is induced by the presence of pre-existing older forms (Smithers and Terry 1969).

Urine egg microscopy demonstrated a low sensitivity, indicating that it failed to detect a large proportion of women classified as infected by the latent class model. This finding is expected in the context of female genital schistosomiasis, where urinary egg excretion may be absent, intermittent, or unrelated to the extent of genital involvement. This means that many women with genital schistosomiasis may not necessarily shed detectable eggs in urine at the time of examination (Colombe et al. 2018). Egg excretion is variable, depends on both the worm load and the host immunity, and can fluctuate on a daily basis (Doehring et al. 1983; Birrie et al. 1994).

Colposcopy identified only a negligible proportion of the women classified as positive by the latent class model. This result suggests that visible genital lesions alone are not adequate for identifying most cases of FGS in this study population. Several explanations may account for this: FGS lesions may not be visible in all infected women and not all women present disease manifestation in the same way. As shown in our study, we observed various combinations of FGS signs and symptoms and lesions maybe subtle or non-specific mimicking other conditions like cervicitis, STIs, inflammation etc. (Leutscher et al. 2008; Shukla et al. 2023; Sturt et al. 2023). Unlike PCR or egg microscopy, colposcopy does not detect the parasite itself. It instead identifies structural or inflammatory consequences of infection, which may occur only in a subset of infected women. Thus, the low sensitivity observed here suggests that lesion-based assessment alone is likely to miss the majority of latent infections and should therefore not be used as a sole diagnostic strategy for FGS. Moreover, visual imaging can be useful in the assessment of Schistosoma related morbidity, praziquantel treatment response, and defining the natural history of visual-FGS (Sturt et al. 2023). 

In the absence of a definitive gold standard for Female Genital Schistosomiasis (FGS), Latent Class Analysis (LCA) provides a valuable framework for integrating information from multiple imperfect diagnostic tests. In this study, the model-estimated latent prevalence was higher than the apparent prevalences obtained by colposcopy and urine egg microscopy, indicating that these individual tests may underestimate the occurrence of FGS when used in isolation. These findings reinforce the need for complementary diagnostic strategies that combine molecular, parasitological, and clinical assessments to improve the identification of women with FGS in endemic settings.

Importantly, the observed differences across diagnostic methods should not necessarily be interpreted as evidence that one test alone is sufficient for the diagnosis of female genital schistosomiasis. Rather, the findings underscore the complementary nature of these approaches. Each method captures a different dimension of disease: colposcopy identifies visible genital lesions (mostly chronic infections) and mucosal pathology, urine egg microscopy reflects active urinary egg excretion (eggs not excreted at every urinary collection point), and PCR detects parasite DNA (molecular detection not evident to the clinicians), which may be present even in the absence of overt lesions or detectable egg shedding. Because these biological and clinical manifestations do not always occur simultaneously, no single diagnostic method is likely to fully characterize all cases. Therefore, the higher positivity observed with PCR may indicate improved detection of otherwise missed infections, while colposcopy and microscopy continue to provide clinically and parasitologically relevant information that cannot be fully replaced by molecular detection alone. Because these manifestations do not necessarily occur simultaneously in the same woman, discordance between tests is expected and does not necessarily imply that one method is “wrong” but reflects the biological heterogeneity of FGS.

In the overall evaluation of the observed gynecological manifestations, blood in urine was significantly associated with positive egg count which also corroborates previous findings (Randrianasolo et al. 2015). Also, other symptoms like joint pains, painful urination and vulvuvaginal itches and homogenous sandy patches in our study population was significantly associated with egg ova which corroborates previous findings by (Kjetland et al. 2005).

Most of our study participants (72.8%) were in the 15–34 years age group. These are active women in the community who are more likely to be involved in day-to-day activities like fetching water, washing dresses which brings them into contact with water from the dam. More than half (53.1%) were housewives which exposes them more to such activities. Interestingly, 87.4% of these women carry out laundry activities in the dam and 77.6% of them rely on water sources from the dam explaining the high probability of exposure to the infection. Interestingly, Fishing, community and region were significantly associated with egg counts as reported in other studies (Masong et al. 2021). This indicates that these women continuously serve as reservoirs of transmission of the disease accounting for the high PCR positivity rate. No treatment was also strongly associated to PCR positivity rate reiterating the need for integrated control strategies including WASH and MDA programs.

Overall, these findings illustrate the inherent limitations of gold-standard-free modeling in the evaluation of FGS diagnostics. The high proportion of PCR-positive cervical swab samples should be interpreted with caution. While detection of the Dra1 repeat indicates the presence of *S. haematobium* DNA within the genital tract, PCR does not distinguish between DNA derived from viable parasites or eggs and residual DNA that may persist following parasite death or reduced egg excretion. Consequently, PCR positivity should not be interpreted as definitive evidence of active infection in isolation. Rather, molecular findings should be considered alongside parasitological and clinical assessments to provide a more comprehensive evaluation of Female Genital Schistosomiasis.

### Strengths and limitations

The sample size was smaller than planned and larger studies would be valuable to further validate these findings. Recruitment was affected by limited participation in some communities due to religious beliefs and reluctance of some women to undergo pelvic examination. In addition, sample size estimation relied on non-local prevalence data for cervical Dra1 probe-based PCR because local estimates were unavailable. The absence of a definitive gold standard for FGS diagnosis also necessitated the use of latent class analysis (LCA). Although LCA is a robust approach for evaluating diagnostic tests without a reference standard, the resulting sensitivity and specificity estimates are model-dependent and should be interpreted accordingly. Furthermore, urine egg microscopy, cervical Dra1 probe-based PCR, and colposcopy detect different biological manifestations of *S. haematobium* infection, which may not fully satisfy the assumption of conditional independence.

Despite these limitations, the study has several important strengths. It represents one of the few evaluations comparing parasitological, molecular, and clinical diagnostic approaches for FGS within a latent class modelling framework. By integrating complementary diagnostic methods, the study provides a more comprehensive assessment of FGS than could be achieved using any single test alone. These findings highlight the value of multimodal diagnostic strategies for improving the detection of both active *S. haematobium* infection and genital disease manifestations, thereby supporting earlier diagnosis, appropriate clinical management, and progress toward improved FGS control in endemic settings.

## Conclusion

In the absence of a definitive gold standard, latent class analysis highlighted the complementary contributions of urine egg microscopy, cervical PCR, and colposcopy to the diagnosis of Female Genital Schistosomiasis. No single diagnostic method adequately captured all aspects of the disease, underscoring the value of combining molecular, parasitological, and clinical approaches to improve disease detection and guide public health interventions in endemic communities.

## Supplementary Information

Below is the link to the electronic supplementary material.

Supplementary Material 1

## Data Availability

The data supporting the findings of this study are not publicly available due to some information that could compromise the privacy of research participants. Requests to access the analytical data should be directed to the authors or the relevant supplementary information.

## Abbreviations

FGS: Female genital schistosomiasis
DNA: Deoxyribonucleic acid
MDA: Mass drug administration
WASH: Water, sanitation and hygiene
PCR: Polymerase chain reaction
WHO: World health organization
OR: Odds ratio
CI: Confidence interval

## References

[CR1] Agnew A, Fulford AJC, Mwanje MT, Gachuhi K, Gutsmann V, Krijger FW, Sturrock RF, Vennervald BJ, Ouma JH, Butterworth AE et al (1996) Age-Dependent Reduction of Schistosome Fecundity in Schistosoma haematobium But Not Schistosoma Mansoni Infections in Humans. Am J Trop Med Hyg 55(3):338–343. 10.4269/ajtmh.1996.55.338 8842126

[CR2] Baltrušis P, Höglund J (2023) Digital PCR: modern solution to parasite diagnostics and population trait genetics. Parasit Vectors 16:143. 10.1186/s13071-023-05756-7 37098569 PMC10131454

[CR3] Birrie H, Medhin G, Erko B (1994) Variability of egg excretion in Schistosoma mansoni infection in Ethiopia: a case report. East Afr Med J 71(8):545–547. PMID 7867551 7867551

[CR4] Bustinduy AL, Randriansolo B, Sturt AS, Kayuni SA, Leutscher PDC, Webster BL, Van Lieshout L, Stothard JR, Feldmeier H, Gyapong M (2022) Chapter One - An update on female and male genital schistosomiasis and a call to integrate efforts to escalate diagnosis, treatment and awareness in endemic and non-endemic settings: The time is now. Rollinson D and Stothard R (eds) Adv Parasitol 115:1–44. 10.1016/bs.apar.2021.12.003 35249661

[CR5] Cheesbrough M (1985) Medical Laboratory Manual for Tropical Countries. ELBS with Tropical Health Technology/Butterworth-Heinemann, Cambridgeshire

[CR6] Clegg JA, Smithers SR, Terry RJ (1971) Concomitant immunity and host antigens associated with schistosomiasis. Int J Parasitol 1(1) :43–49 10.1016/0020-7519(71)90045-2 5005564

[CR7] Cnops L, Soentjens P, Clerinx J, Van Esbroeck M (2013) A Schistosoma haematobium-Specific Real-Time PCR for Diagnosis of Urogenital Schistosomiasis in Serum Samples of International Travelers and Migrants. PLoS Negl Trop Dis 7(8):e2413. 10.1371/journal.pntd.0002413 24009791 PMC3757062

[CR8] Colley DG, Bustinduy AL, Secor WE, King CH (2014) Human schistosomiasis. Lancet (London England) 383(9936):2253–2264. 10.1016/S0140-6736(13)61949-2 24698483 PMC4672382

[CR9] Colombe S, Lee MH, Masikini PJ, van Lieshout L, de Dood CJ, Hoekstra PT, Corstjens PLAM, Mngara J, van Dam GJ, Downs JA (2018) Decreased Sensitivity of Schistosoma sp. Egg Microscopy in Women and HIV-Infected Individuals. Am J Trop Med Hyg 98(4):1159–1164. 10.4269/ajtmh.17-0790 29405114 PMC5928829

[CR10] Dendukuri N, Joseph L (2001) Bayesian Approaches to Modeling the Conditional Dependence Between Multiple Diagnostic Tests. Biometrics 57:158–167. 10.1111/j.0006-341X.2001.00158.x 11252592

[CR11] Doehring E, Feldmeier H, Daffalla AA (1983) Day-to-day variation and circadian rhythm of egg excretion in urinary schistosomiasis in the Sudan. Ann Trop Med Parasitol 77(6):587–594. 10.1080/00034983.1983.11811757 6660966

[CR41] Hamburger J, Turetski T, Kapeller I, Deresiewicz R (1991) Highly repeated short DNA sequences in the genome of Schistosoma mansoni recognized by a species-specific probe. Mol Biochem Parasitol 44 (1):73-80. 10.1016/0166-6851(91)90222-R 2011155

[CR12] Hui SL, Walter SD (1980) Estimating the error rates of diagnostic tests. Biometrics 36(1):167–171. 10.2307/2530508 7370371

[CR13] Ibironke O, Koukounari A, Asaolu S, Moustaki I, Shiff C (2012) Validation of a New Test for Schistosoma haematobium Based on Detection of Dra1 DNA Fragments in Urine: Evaluation through Latent Class Analysis. PLoS Negl Trop Dis 6(1):e1464. 10.1371/journal.pntd.0001464 22235360 PMC3250497

[CR14] Joseph L, Gyorkos TW, Coupal L (1995) Bayesian Estimation of Disease Prevalence and the Parameters of Diagnostic Tests in the Absence of a Gold Standard. Am J Epidemiol 141(3):263–272. 10.1093/oxfordjournals.aje.a117428 7840100

[CR15] Keddie SH, Baerenbold O, Keogh RH, Bradley J (2023) Estimating sensitivity and specificity of diagnostic tests using latent class models that account for conditional dependence between tests: a simulation study. BMC Med Res Methodol 23:58. 10.1186/s12874-023-01873-0 36894883 PMC9999546

[CR16] Kjetland EF, Ndhlovu PD, Mduluza T, Gomo E, Gwanzura L, Mason PR, Kurewa EN, Midzi N, Friis H, Gundersen SG (2005) Simple clinical manifestations of genital Schistosoma haematobium infection in rural Zimbabwean women. Am J Trop Med Hyg 72(3):311–319. 10.4269/ajtmh.2005.72.311 15772328

[CR17] Kjetland EF, Leutscher PD, Ndhlovu PD (2012) A review of female genital schistosomiasis. Trends Parasitol 28(2):58–65. 10.1016/j.pt.2011.10.008 22245065

[CR18] Kjetland EF, Norseth HM, Taylor M, Lillebø K, Kleppa E, Holmen SD, Andebirhan A, Yohannes TH, Gundersen SG, Vennervald BJ et al (2014) Classification of the lesions observed in female genital schistosomiasis. Int J Gynecol Obstet 127:227–228. 10.1016/j.ijgo.2014.07.014 25179171

[CR19] Landis JR, Koch GG (1977) The measurement of observer agreement for categorical data. Biometrics 33(1):159–174. 10.2307/2529310 843571

[CR20] Leutscher PDC, Ramarokoto C-E, Hoffmann S, Jensen JS, Ramaniraka V, Randrianasolo B, Raharisolo C, Migliani R, Christensen N (2008) Coexistence of Urogenital Schistosomiasis and Sexually Transmitted Infection in Women and Men Living in an Area Where Schistosoma haematobium is Endemic. Clin Infect Dis 47(6):775–782. 10.1086/591127 18680415

[CR21] Makia CM, Fesuh NB, Amabo EN, Gamba VA, Oluwole AS, Stothard R (2023) Urogenital schistosomiasis (UGS) and female genital schistosomiasis (FGS) in Cameroon: an observational assessment of key reproductive health determinants of girls and women in the Matta Health Area. BMJ Open 13:e063392. 10.1136/bmjopen-2022-063392 36787976 PMC9930553

[CR22] Masong MC, Wepnje GB, Marlene NT, Gamba V, Mengue M-T, Kouokam E, Stothard JR, Ekobo ALS (2021) Female Genital Schistosomiasis (FGS) in Cameroon: A formative epidemiological and socioeconomic investigation in eleven rural fishing communities. PLoS Glob Public Health 1(10):e0000007. 10.1371/journal.pgph.0000007 36962084 PMC10022362

[CR23] Mberu M, Zongo K, Leaning E, Makau-Barasa L, Kamara K, Jacobson J (2025) Female genital schistosomiasis: Addressing diagnostic and programmatic gaps to advance elimination efforts. Int J Infect Dis 152:107798. 10.1016/j.ijid.2025.107798 39870160

[CR24] Mewabo AP, Moyou RS, Kouemeni LE, Ngogang JY, Kaptue L, Tambo E (2017) Assessing the prevalence of urogenital schistosomaisis and transmission risk factors amongst school-aged children around Mapé dam ecological suburbs in Malantouen district, Cameroon. Infect Dis Poverty 6(1):40. 10.1186/s40249-017-0257-7 28260525 PMC5338087

[CR25] Naing L, Nordin RB, Abdul Rahman H, Naing YT (2022) Sample size calculation for prevalence studies using Scalex and ScalaR calculators. BMC Med Res Methodol 22:209. 10.1186/s12874-022-01694-7 35907796 PMC9338613

[CR26] Njunda AL, Ndzi EN, Assob JCN, Kamga H-LF, Kwenti ET (2017) Prevalence and factors associated with urogenital schistosomiasis among primary school children in barrage, Magba sub-division of Cameroon. BMC Public Health 17:618. 10.1186/s12889-017-4539-6 28673343 PMC5496429

[CR27] Norseth HM, Ndhlovu PD, Kleppa E, Randrianasolo BS, Jourdan PM, Roald B, Holmen SD, Gundersen SG, Bagratee J, Onsrud M et al (2014) The Colposcopic Atlas of Schistosomiasis in the Lower Female Genital Tract Based on Studies in Malawi, Zimbabwe, Madagascar and South Africa. PLoS Negl Trop Dis 8(11):e3229. 10.1371/journal.pntd.0003229 25412334 PMC4238986

[CR40] Poggensee G, Hermann Feldmeier (2001) Female genital schistosomiasis: facts and hypothesis. Acta Tropica 79:193-210. 10.1016/S0001-706X(01)00086 11412803

[CR28] Rachid T, Abarda A, Hasbaoui A (2024) Latent class analysis: A review and recommendations for future applications in health sciences. Procedia Comput Sci 238:1062–1067. 10.1016/j.procs.2024.06.135

[CR29] Randrianasolo BS, Jourdan PM, Ravoniarimbinina P, Ramarokoto CE, Rakotomanana F, Ravaoalimalala VE, Gundersen SG, Feldmeier H, Vennervald BJ, van Lieshout L et al (2015) Gynecological Manifestations, Histopathological Findings, and Schistosoma-Specific Polymerase Chain Reaction Results Among Women With Schistosoma haematobium Infection: A Cross-sectional Study in Madagascar. J Infect Dis 212(2):275–28. 10.1093/infdis/jiv035 25725656 PMC4482143

[CR30] Shukla JD, Kleppa E, Holmen S, Ndhlovu PD, Mtshali A, Sebitloane M, Vennervald BJ, Gundersen SG, Taylor M, Kjetland EF (2023) The Association Between Female Genital Schistosomiasis and Other Infections of the Lower Genital Tract in Adolescent Girls and Young Women: A Cross-Sectional Study in South Africa. J Low Genit Tract Dis 27(3):291–296. 10.1097/LGT.0000000000000756 37379442 PMC10309100

[CR31] Smithers SR, Terry RJ (1969) Immunity in Schistosomiasis. Ann N Y Acad Sci 160(2):826–840. 10.1111/j.1749-6632.1969.tb15904.x 4981050

[CR32] Sturt A, Bristowe H, Webb E, Hansingo I, Phiri C, Mudenda M, Mapani J, Mweene T, Levecke B, Cools P et al (2023) Visual diagnosis of female genital schistosomiasis in Zambian women from hand-held colposcopy: agreement of expert image review and association with clinical symptoms. Wellcome Open Res 8:14. 10.12688/wellcomeopenres.18737.2 36864924 PMC9971661

[CR33] Tchuem Tchuenté L-A, Rollinson D, Stothard JR, Molyneux D (2017) Moving from control to elimination of schistosomiasis in sub-Saharan Africa: time to change and adapt strategies. Infect Dis Poverty 6:42. 10.1186/s40249-017-0256-8 28219412 PMC5319063

[CR34] Utzinger J, Becker SL, Van Lieshout L, Van Dam GJ, Knopp S (2015) New diagnostic tools in schistosomiasis. Clin Microbiol Infect 21(6):529–542. 10.1016/j.cmi.2015.03.014 25843503

[CR35] Valls J, Baena A, Venegas G, Celis M, González M, Sosa C, Santin JL, Ortega M, Soilán A, Turcios E et al (2023) Performance of standardised colposcopy to detect cervical precancer and cancer for triage of women testing positive for human papillomavirus: results from the ESTAMPA multicentric screening study. Lancet Glob Health 11(3):e350–e360 36796982 10.1016/S2214-109X(22)00545-9 PMC10020136

[CR42] Wall KM, Kilembe W, Vwalika B, Dinh C, Livingston P, Lee YM, Lakhi S, Boeras D, Naw HK, Brill I, Chomba E, Sharkey T, Parker R, Shutes E, Tichacek A, Secor WE, Allen S. (2018) Plos Neg Trop Dis 12(12):e0006902. 10.1371/journal.pntd.0006902 PMC6292564 30543654

[CR36] Weerakoon KGAD, Gobert GN, Cai P, McManus DP (2015) Advances in the Diagnosis of Human Schistosomiasis. Clin Microbiol Rev 28(4):939–967. 10.1128/cmr.00137-14 26224883 PMC4548261

[CR37] WHO (2002) Tropical Disease Research, TDR Strategic Direction: Schistosomiasis. WHO-TDR, Geneva

[CR38] WHO (2015) Female Genital Schistosomiasis: A Pocket Atlas for Clinical Health Care Professionals. World Health Organization, Geneva

[CR39] WHO (2022) Guideline on control and elimination of human schistosomiasis. World Health Organization, Geneva 35235279

