# Supplementary material for: Molecular, parasitological and clinical diagnosis of female genital schistosomiasis: A diagnostic yield and comparative accuracy study using latent class analysis
Source: Parasitol Res. 2026 Sep 18;125(1):111. doi: 10.1007/s00436-026-08753-8 (PMC13597625; doi:10.1007/s00436-026-08753-8)
Supplement: Supplementary file 1 [file 436_2026_8753_MOESM1_ESM.pdf]

Molecular, Parasitological and Clinical Diagnosis of Female Genital Schistosomiasis: A Diagnostic Yield and Comparative Accuracy study using Latent Class Analysis

Figure 1: Melt Curve Analysis for *Schistosoma haematobium q*PCR

"Quantitative PCR was performed to detect the Schistosoma haematobium Dra genes. Melt curve analysis showed a single peak ranging from 77 °C to 80 °C across all samples, consistent with the predicted melting temperature of the target amplicon. No additional distinct peaks were observed, confirming the specificity of the amplification."

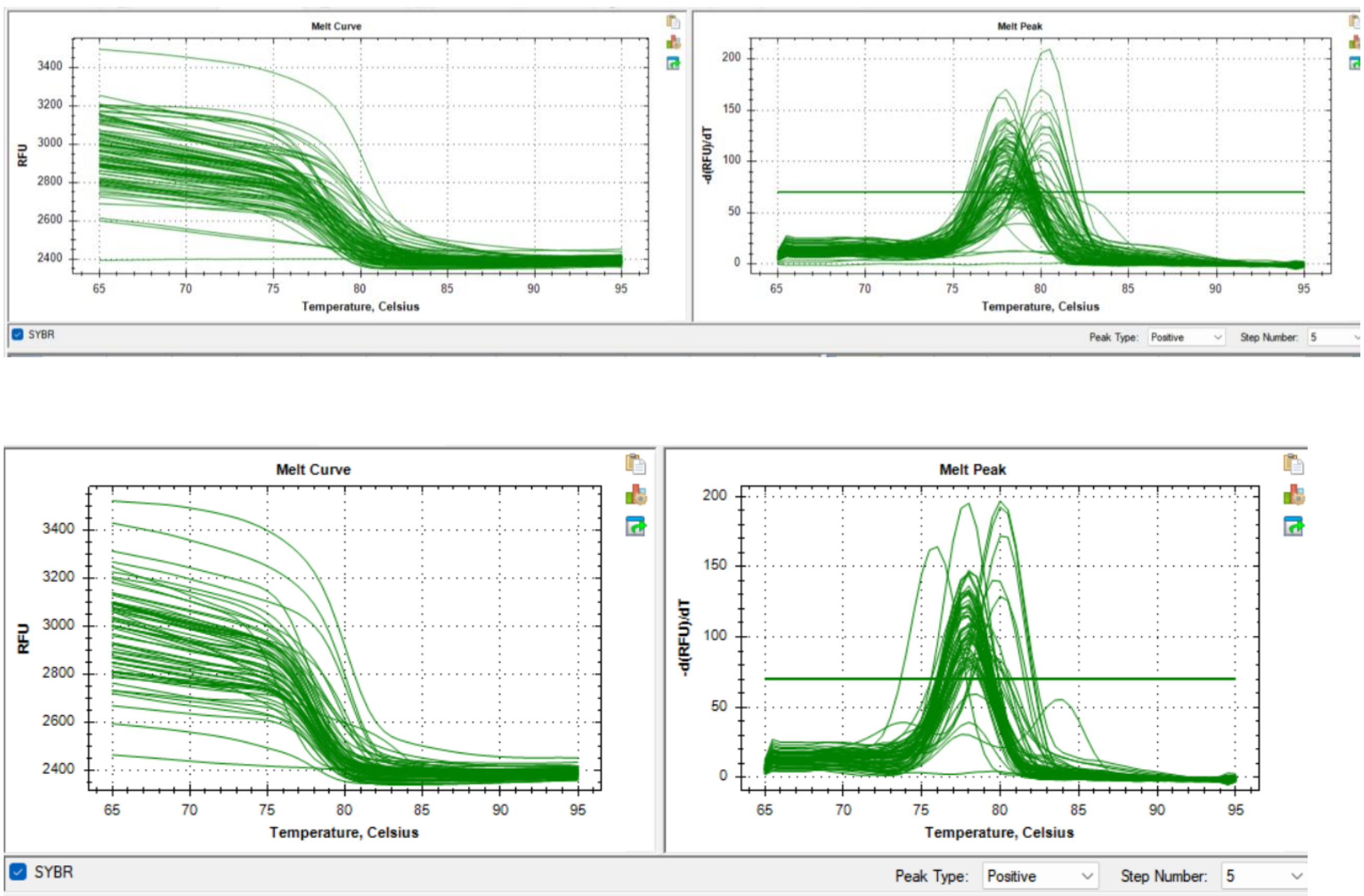

Figure 2: Probe based assays (Probe 5'-6FAM TGTTGGTGGAAGTGCCTGTTTCGCAA-MGB- 3') and agarose gel electrophoresis to confirm the specificity of the PCR data generated in the probe experiment

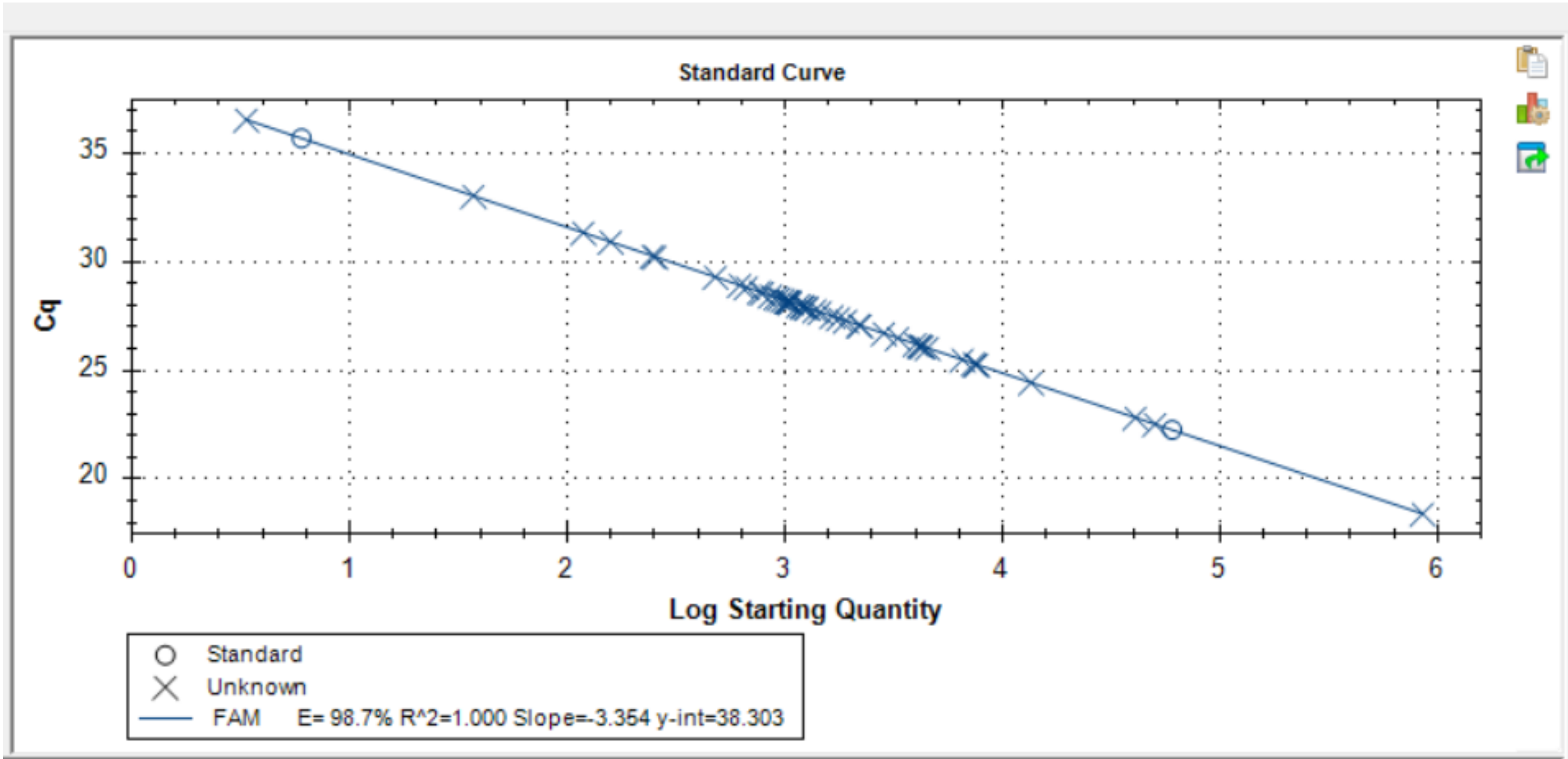

A) PCR Efficiency of Probe based PCR

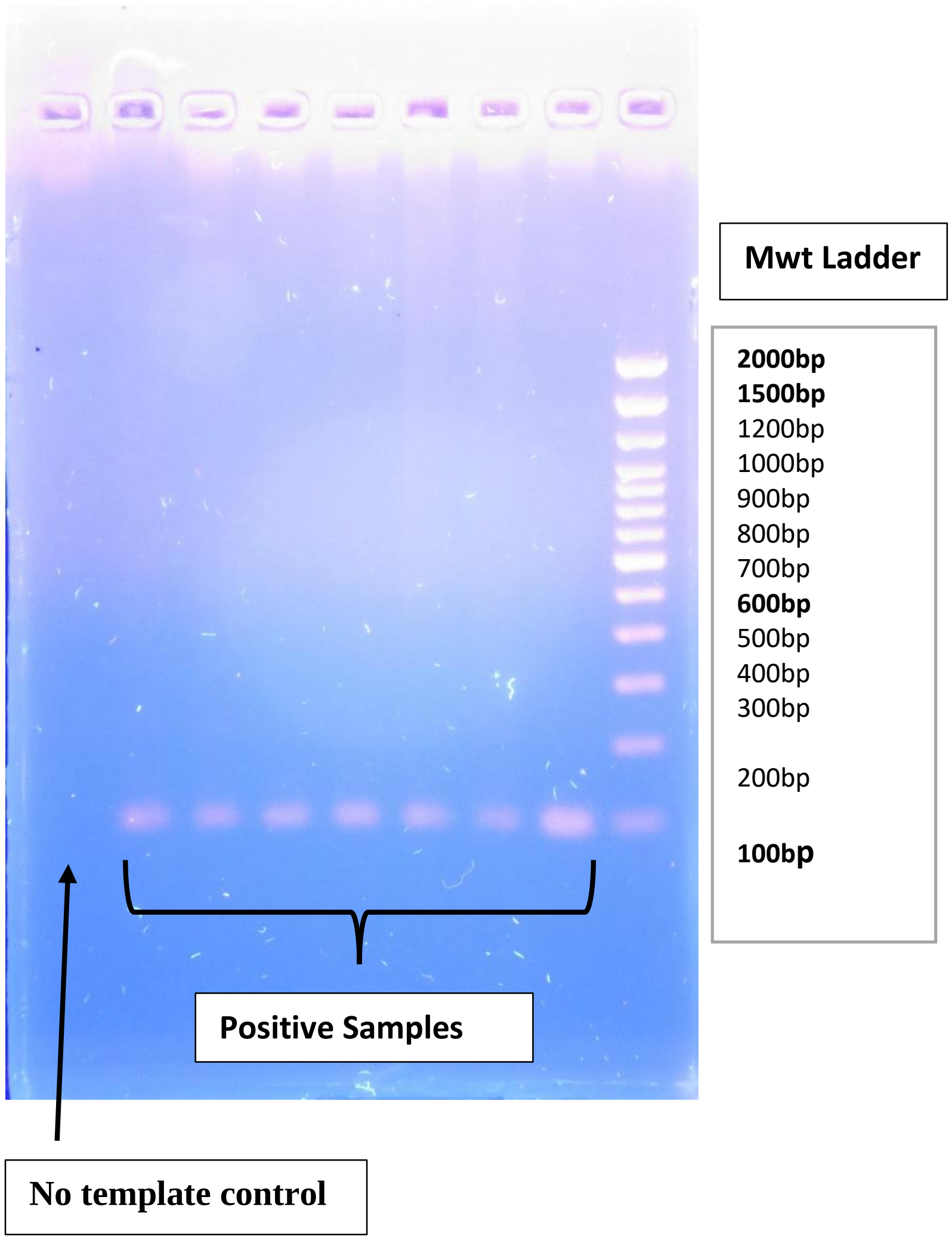

B) Gel 1: Electrophoregram indicating positive and negative participant samples (discriminative ability of the PCR Tests)

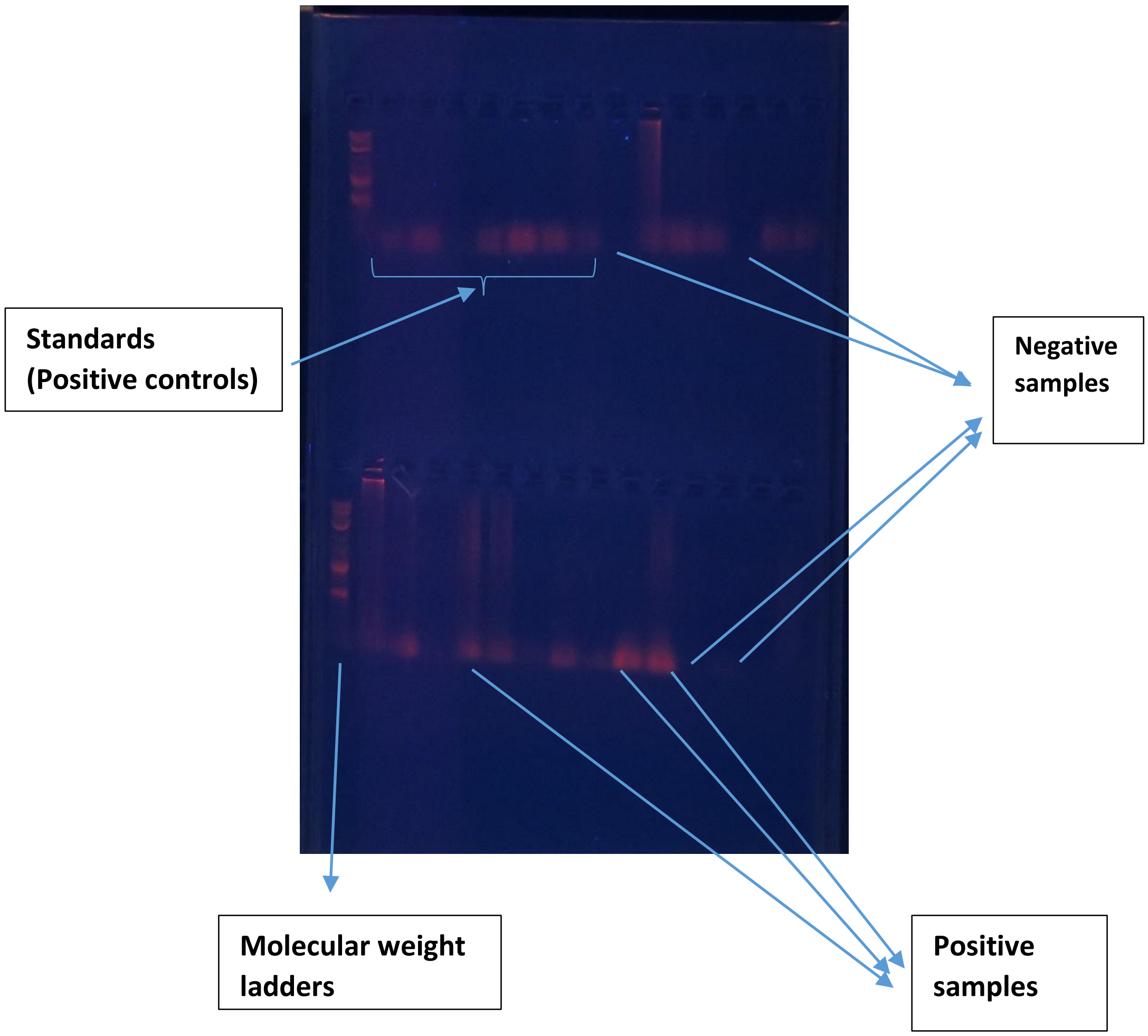

C) Gel 2: Electrophoregram indicating positive and negative participant samples (discriminative ability of the PCR Tests)

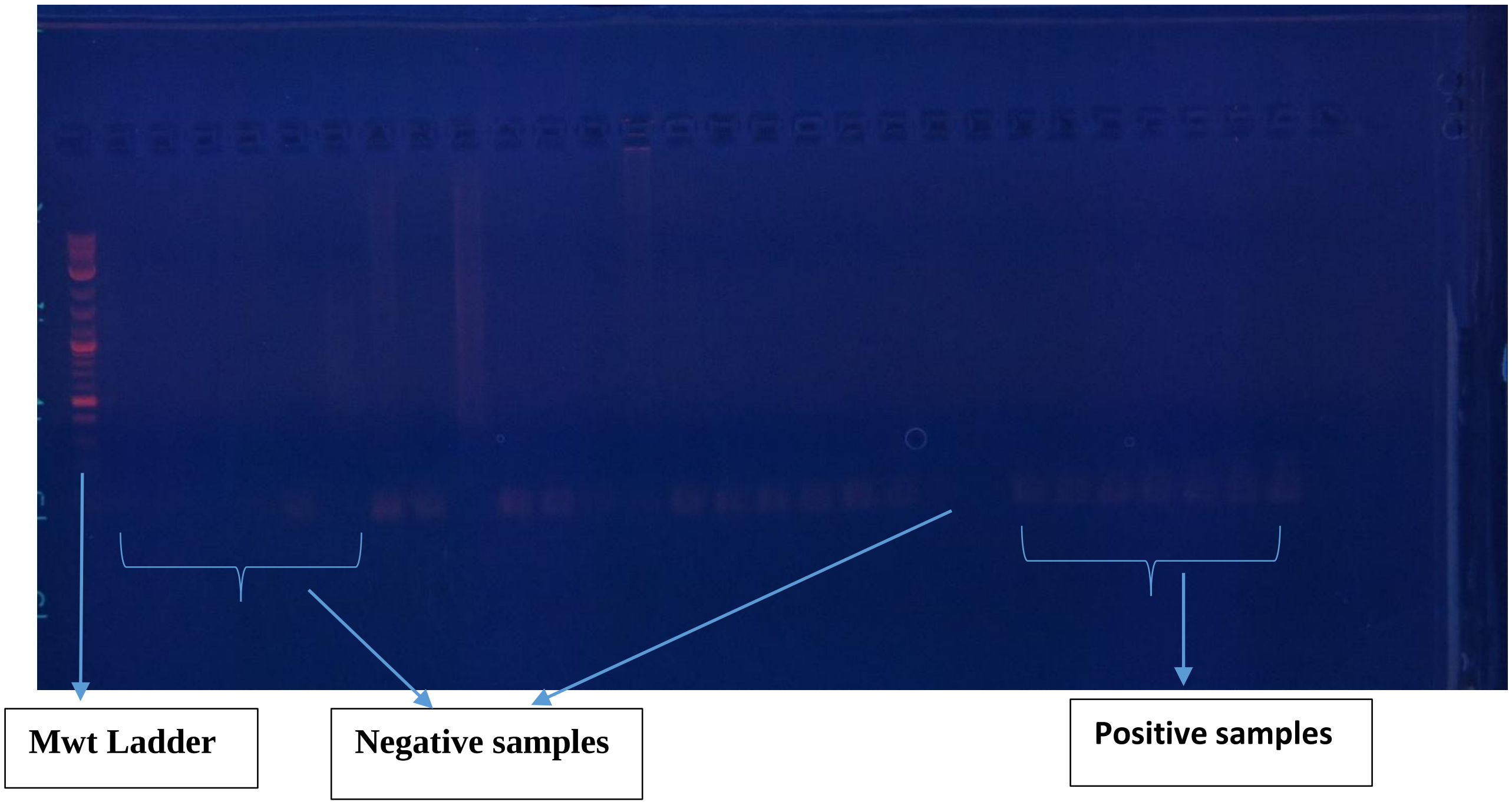

D) Gel 3: Electrophoregram indicating positive and negative participant samples (discriminative ability of the PCR experiment)

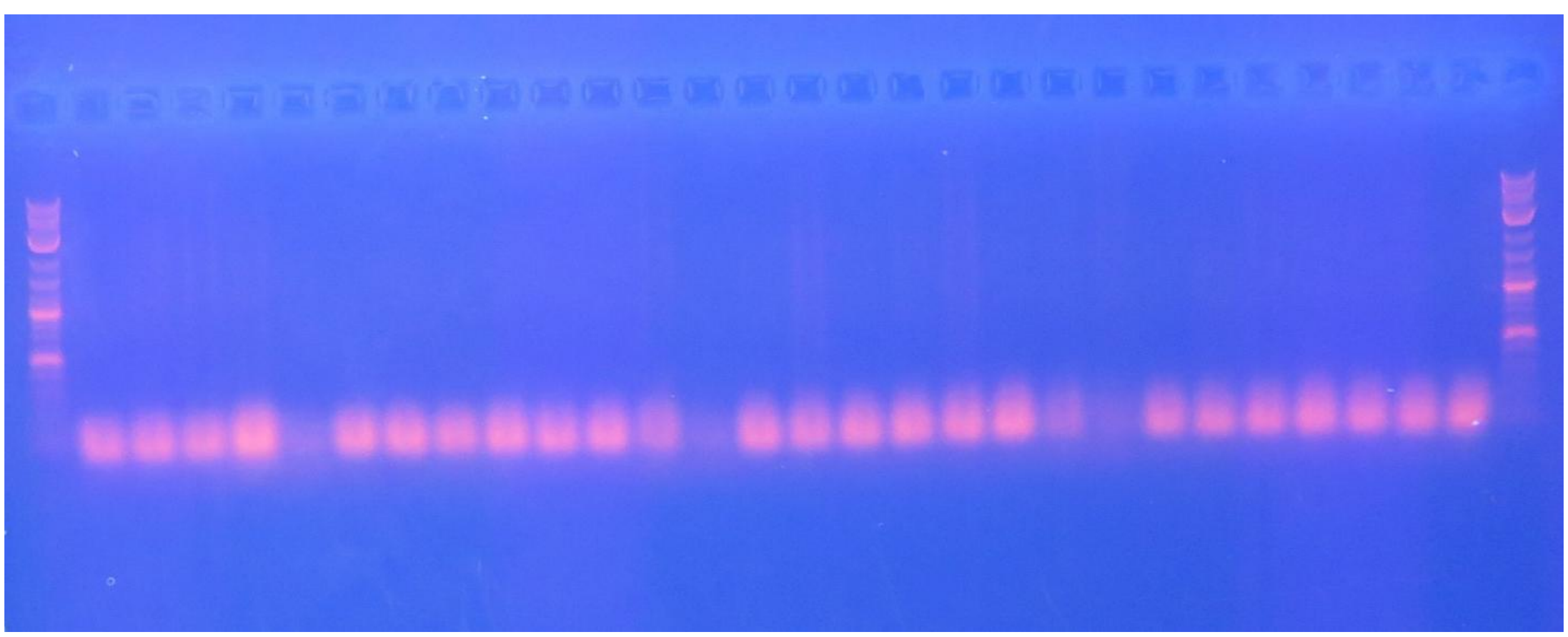

E) Gel 4: Electrophoregram indicating positive and negative participant samples (discriminative ability of the PCR Tests)

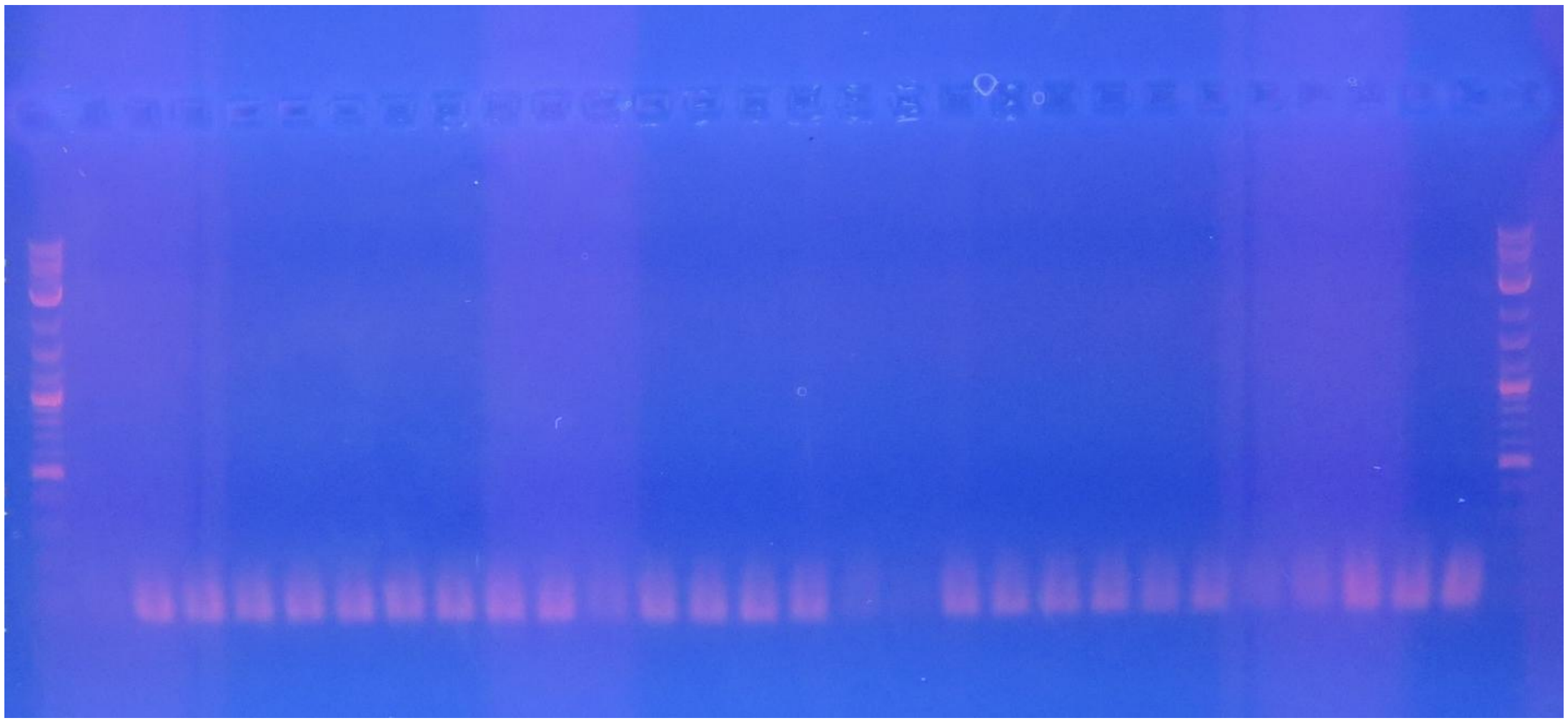

F) Gel 5: Electrophoregram indicating positive and negative participant samples (discriminative ability of the PCR Tests)

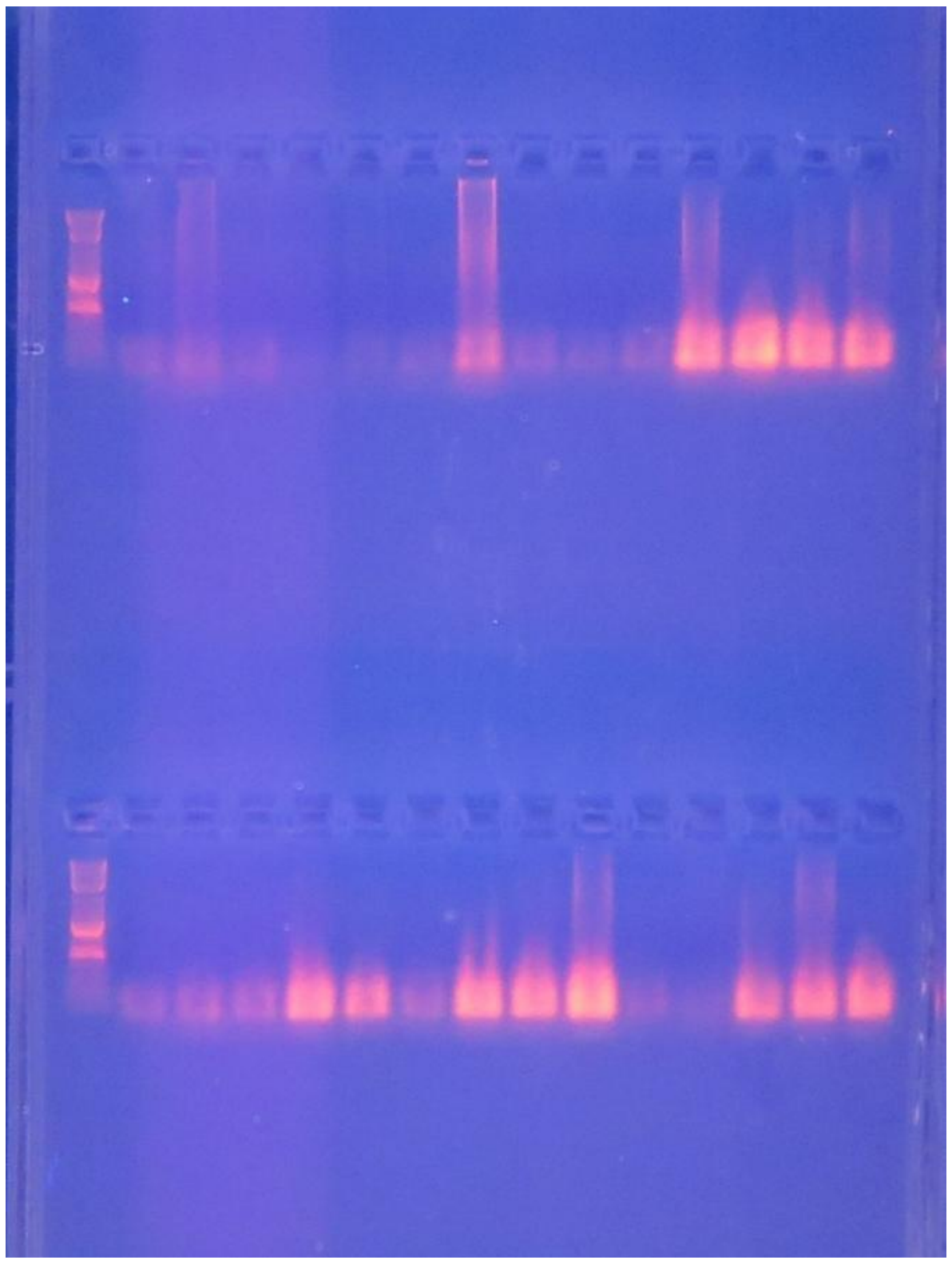

G) Gel 6: Electrophoregram indicating positive and negative participant samples (discriminative ability of the PCR Tests)
